# Impact of Quorum Sensing and Tropodithietic Acid Production on the Exometabolome of *Phaeobacter inhibens*

**DOI:** 10.3389/fmicb.2022.917969

**Published:** 2022-06-21

**Authors:** Sujatha Srinivas, Martine Berger, Thorsten Brinkhoff, Jutta Niggemann

**Affiliations:** Institute for Chemistry and Biology of the Marine Environment, University of Oldenburg, Oldenburg, Germany

**Keywords:** exometabolome, quorum sensing, tropodithietic acid, *Phaeobacter*, mutants, secondary metabolites, FT-ICR-MS, microbial interactions

## Abstract

Microbial interactions shape ecosystem diversity and chemistry through production and exchange of organic compounds, but the impact of regulatory mechanisms on production and release of these exometabolites is largely unknown. We studied the extent and nature of impact of two signaling molecules, tropodithietic acid (TDA) and the quorum sensing molecule acyl homoserine lactone (AHL) on the exometabolome of the model bacterium *Phaeobacter inhibens* DSM 17395, a member of the ubiquitous marine *Roseobacter* group. Exometabolomes of the wild type, a TDA and a QS (AHL-regulator) negative mutant were analyzed *via* Fourier-transform ion cyclotron resonance mass spectrometry (FT-ICR-MS). Based on a total of 996 reproducibly detected molecular masses, exometabolomes of the TDA and QS negative mutant were ∼70% dissimilar to each other, and ∼90 and ∼60% dissimilar, respectively, to that of the wild type. Moreover, at any sampled growth phase, 40–60% of masses detected in any individual exometabolome were unique to that strain, while only 10–12% constituted a shared “core exometabolome.” Putative annotation revealed exometabolites of ecological relevance such as vitamins, amino acids, auxins, siderophore components and signaling compounds with different occurrence patterns in the exometabolomes of the three strains. Thus, this study demonstrates that signaling molecules, such as AHL and TDA, extensively impact the composition of bacterial exometabolomes with potential consequences for species interactions in microbial communities.

## Introduction

Microorganisms release a complex blend of primary and secondary metabolites into their environment, collectively termed as exometabolome, depending on nutrient regimes, environmental cues and physiological state (Kell et al., 2005; Villas-Bôas et al., 2006; Romano et al., 2014; Wienhausen et al., 2017). In addition to well-known exometabolites such as those involved in nutrient acquisition [e.g., siderophores (Mansson et al., 2011; Walker et al., 2017)], communication [e.g., acyl homoserine lactones, pheromones (Gram et al., 2002; Atkinson and Williams, 2009; Gillard et al., 2013)], antagonism [e.g., antibiotics (Thole et al., 2012)], mutualism [e.g., vitamins, auxins (Buchan et al., 2014; Sañudo-Wilhelmy et al., 2014)] or chemical defense [e.g., polyunsaturated aldehydes (Wichard et al., 2005)], a wealth of metabolites, many with so far unknown structural identities and functions are also released (Rosselló-Mora et al., 2008; Barofsky et al., 2009; Kujawinski et al., 2009; Becker et al., 2014; Romano et al., 2014; Fiore et al., 2015; Longnecker et al., 2015; Wienhausen et al., 2017). This becomes evident using ultra-high resolution untargeted approaches like Fourier-transform ion cyclotron resonance mass spectrometry (FT-ICR-MS), which reveals a metabolic fingerprint of exudates on molecular formula level. Some of these unknown compounds might originate from overflow metabolism (Paczia et al., 2012) or excretion of waste products (Lawrence et al., 2012; Hom and Murray, 2014). However, considering the metabolites detected in exometabolomes of various single organisms and in environmental samples, it is likely that the majority of exometabolites have an ecological function, as in the case of auxotrophs and helpers, host-associations and mixed biofilms.

The *Roseobacter* group within the *Alphaproteobacteria* is a highly abundant and globally distributed marine lineage, containing the largest proportion (72%) of described genera in this class (Buchan et al., 2005; Wagner-Döbler and Biebl, 2006; Brinkhoff et al., 2008; Luo and Moran, 2014; Pohlner et al., 2019). *Phaeobacter inhibens* is a well investigated model organism of the *Roseobacter* group and a prolific biofilm former, capable of colonizing biotic and abiotic surfaces (Seyedsayamdost et al., 2011; Thole et al., 2012; Prol García et al., 2014; Gram et al., 2015; Segev et al., 2016). Besides ecologically relevant metabolites, like algicides and siderophores, *P. inhibens* produces several tropone products including tropodithietic acid (TDA), which serves as both antibiotic (Liang, 2003; Brinkhoff et al., 2004; Porsby et al., 2011; Rabe et al., 2014) and quorum sensing (QS) signal and therefore as a global gene regulator, as shown by the transcriptomic analysis of *P. inhibens* (Beyersmann et al., 2017). Many roseobacters are capable of *N*-acyl-homoserine lactone (AHL)-mediated QS wherein the AHL binds to a LuxR-type transcriptional regulator, thus controlling gene expression often on a global scale and in a cell density-dependent manner (Cude and Buchan, 2013; Patzelt et al., 2013; Zan et al., 2014; Gao et al., 2016). QS-regulated phenotypes include motility and chemotaxis (Wheeler et al., 2006; Patzelt et al., 2013; Zan et al., 2015), biofilm formation (Miller and Bassler, 2001; Zan et al., 2012), intra- and extracellular metabolite production (Latifi et al., 1995; Berger et al., 2011; Cude et al., 2012; Johnson et al., 2016) and horizontal gene transfer (Antonova and Hammer, 2011; Blokesch, 2012; Patterson et al., 2016; Zhu et al., 2020). In *P. inhibens*, biosynthesis of TDA is also regulated by AHL-mediated QS, however, TDA can autoinduce its own synthesis by inducing expression of *tdaA*, which encodes a transcriptional activator for the *tda* genes. At sub-inhibitory concentrations, the TDA molecule can interact with the LuxR-type transcriptional regulator PgaR, mediate QS and influence expression of ∼10% of the total genes, in the same manner as the AHL. Furthermore, absence of TDA leads to decreased surface attachment and cell motility, thus indicating that TDA-based QS mediates the switch from pelagic to sessile lifestyle for *P. inhibens* (Berger et al., 2011; Beyersmann et al., 2017). *P. inhibens* is not only found in association with eukaryotes, but is also known to control the bacterial community assembly on the marine diatom *Thalassiosira rotula* (Majzoub et al., 2019). Bacterial community composition in *T. rotula* mesocosms in the presence of *P. inhibens* were less diverse and different to those exposed to seawater alone. Closely related taxa were among the most significantly impacted in a co-culture with *P. inhibens* while for a mutant strain with reduced inhibition, almost no differences were observed. This suggests that bacterial signaling rather than antagonism is important for the observed influence of *P. inhibens* on the microbiome assembly (Majzoub et al., 2019).

QS regulation of pathways for organic matter degradation, nutrient acquisition, colonization processes and microbial interactions has been studied extensively in marine bacteria (Urvoy et al., 2022). Previous studies have also discussed the influence of QS in regulating production, release and/or uptake of specific metabolites (An et al., 2014; Johnson et al., 2016; Landa et al., 2017; McRose et al., 2018). Furthermore, QS and/or TDA mutants have been used to analyze the impact of AHLs and TDA on growth dynamics and physiological mechanisms such as cell motility, biofilm formation and morphological heterogeneity (Patzelt et al., 2013; Beyersmann et al., 2017; Will et al., 2017). However, little is known about the impact of these signaling molecules on the full suite of exometabolites released by bacteria. We investigated the impact of AHL and TDA on the exometabolome of *P. inhibens* DSM 17395, by comparing two mutant strains with the wild type (WT). In one of the mutants, the AHL regulator gene *pgaR*, a homolog to the *luxR* gene in *Vibrio fischeri*, was knocked out. Thus, strain *pgaR*^–^ lacks a functional AHL receptor and is subsequently devoid of *N*-3-hydroxydecanoyl-homoserine lactone-mediated QS (Berger et al., 2011; Beyersmann et al., 2017). The second mutant strain *tdaE*^–^ cannot produce TDA due to the inactivation of *tdaE* gene (Thole et al., 2012). We hypothesized a substantial change in the exometabolome of both mutants compared to the WT. We also hypothesized that the exometabolome of *tdaE*^–^ is more similar to the WT since this mutant retains AHL-based QS. Furthermore, we suspected a differential release of specific exometabolites by WT and mutants, as a consequence of the functioning/interruption of a major regulatory mechanism, which would potentially affect the nature of interactions within microbial communities.

## Materials and Methods

### Strains and Genetic Manipulations

Strains used in this study were *Phaeobacter inhibens* DSM 17395 wild type (WT), *tdaE*^–^ mutant strain WP14 (DSM 17395 AFO93376::EZTn5, Gm^r^) (Thole et al., 2012) and *pgaR*^–^ mutant strain WP52 (DSM 17395 AFO90122::EZTn5, Gm^r^) (Berger et al., 2011). In the present study, we performed a complementation assay for strain WP14 (*tdaE*^–^) as WP52 was previously complemented (Berger et al., 2011). Amplification of the *tdaE* gene (PGA1_262p00940) plus ∼1.5 kb flanking regions was performed using the primer pair 5′- AAGAGTTGGCCCTGATCGTG -3′ and 5′- TTTG AGCGCTGCCTTGATCT -3′. The resulting amplicon was ligated into the Eco53kI site of pBBR1MCS-2 using T4-DNA ligase and the ligation mixture was introduced into electrocompetent *E. coli* ST18 cells (Dower et al., 1988; Thoma and Schobert, 2009) (Supplementary Methods). Transformants were selected on half MB plates supplemented with 50 μg/mL 5-aminolevulinic acid (ALA) and 50 μg/mL kanamycin (Km_50_). The complementing plasmid pBBR1MCS2-*tdaE* was transferred from *E. coli* ST18 into *P. inhibens* WP14 mutant by conjugation (Piekarski et al., 2009) (Supplementary Methods). The transconjugants were confirmed by PCR amplification and recovery of wild type phenotype was confirmed by measuring pigmentation and antimicrobial activity (Supplementary Figure 1) according to the method of Berger et al. (2011).

### Cultivation and Sampling

The *P. inhibens* strains were initially grown in Marine Broth 2216 medium (MB; BD Biosciences, Franklin Lakes, NJ, United States), then repeatedly transferred (5x) in artificial seawater (ASW) medium (Zech et al., 2009), modified by excluding EDTA from the trace element solution (Wienhausen et al., 2017) and with 5 mM glucose as sole carbon source (28°C, 100 rpm). After 5 transfers, bacteria were inoculated with a starting OD_600_ of 0.01 in 500 mL ASW medium with 5 mM glucose in quadruplicate 2 L Erlenmeyer flasks (28°C, 100 rpm). Growth was monitored by OD_600_ measurements. Triplicate flasks with ASW with 5 mM glucose were run as sterile blank controls. All plastic and glassware used was rinsed with acidified ultrapure water (Milli-Q, pH 2) and all glassware additionally combusted at 500°C for 3 h.

**FIGURE 1:**
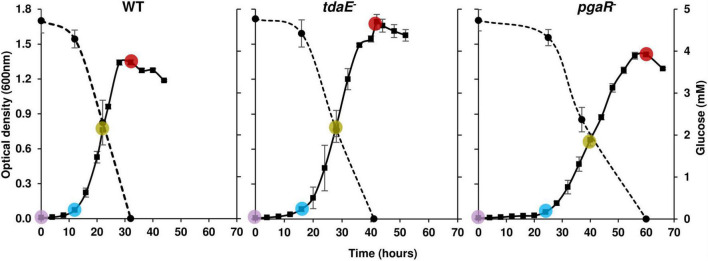
Substrate utilization and growth curves of *P. inhibens* DSM 17395 wild type (WT), *tdaE*^**–**^ mutant and *pgaR*^**–**^ mutant based on optical density on the left Y-axis over time on the X-axis. Solid lines correspond to OD_600_ (left side Y-axis) and dotted lines to glucose concentration (right side Y-axis). Error bars depict standard deviation of four biological replicates. The colored circles correspond to the sampling time points T_0_ (purple), T_1_ (blue), T_2_ (green) and T_3_ (red) for exometabolome analysis of each strain. OD_600_ was measured every 3–4 h.

Subsamples were withdrawn from the cultures for separate analysis of dissolved organic carbon (DOC) (10 mL), dissolved organic matter (DOM) (20 mL), dissolved free (DFAA) (10 mL) and total hydrolysable dissolved amino acids (THDAA) (10 mL), dissolved free neutral monosaccharides (DFNCHO) (10 mL) and dissolved combined monosaccharides (DCNCHO) (10 mL). 70 mL of culture from each flask and at each time point was centrifuged (10 min, 2,499 × *g*, 4°C), supernatants filtered through a 0.22 μm polyethersulfone membrane (Minisart, Sartorius, Göttingen, Germany) and stored in combusted glass vials at −20°C until further analyses. Every biological replicate was sampled immediately after inoculation (T_0_), lag phase (T_1_), mid-exponential (T_2_) and early stationary phase (T_3_). The sampling time points were decided based on OD_600_ values to compare the exometabolomes of the strains when they are in the same growth phase, irrespective of how much time it took them to reach that phase (Figure 1).

### Analysis of Amino Acids, Mono- and Polysaccharides and Dissolved Organic Matter

Concentrations of DFAA and THDAA were analyzed by high performance liquid chromatography (HPLC) after precolumn derivatization with orthophtaldialdehyde (Lunau et al., 2006), and concentrations of DFNCHO and DCNCHO by HPLC and pulsed amperometric detection after desalting (Hahnke et al., 2013). Detection limits for individual amino acids and monosaccharides were 5 nM and 0.5 μM, respectively. However, relatively high concentrations of glucose as a single substrate source interfered with the analysis of mono- and polysaccharides. Dissolved organic carbon in the filtrates of the bacterial cultures and of the solid-phase extracted DOM was quantified after high-temperature catalytic oxidation (Osterholz et al., 2014).

For FT-ICR-MS analyses of DOM, filtrates were acidified to pH 2 (HCl 25% p.a., Carl Roth, Germany), extracted *via* Priority PolLutant (PPL) solid phase cartridges (100 mg; Agilent, Waldbronn, Germany) and resulting extracts adapted to a concentration of 3 ppm carbon in a 1:1 mixture of methanol and ultrapure water (Osterholz et al., 2015). The extraction efficiency increased from 1 to 39% on carbon basis during the course of the experiment, mainly reflecting the decrease in substrate concentration as glucose was not retained on the PPL resin. Extracted DOM was ionized by soft electrospray ionization (ESI; Bruker Apollo, Daltonics, Bremen, Germany) and analyzed in negative mode with a 15 T Solarix FT-ICR-MS (Bruker, Daltonics, Bremen, Germany). For each spectrum, 400 scans were accumulated with an ion accumulation time of 0.1 s in the mass window of 92–1,000 Da. An internal calibration list was generated using Bruker Daltonic Data Analysis software for the calibration of the mass spectra, using at least 20 calibration points. All linear calibrations resulted in an average mass error of 0.05 ppm. Instrument performance and calibration accuracy was monitored by repeated analysis of a laboratory-internal deep ocean DOM reference sample (mass accuracy of less than 0.1 ppm). The samples were measured manually and in a random order. Blank checks with methanol/ultrapure water 1:1 were performed at random intervals.

### Data Processing, Statistical Analysis and Exometabolite Prediction

The detected molecular masses were processed by applying a customized routine Matlab script implemented in ICBM-OCEAN (Merder et al., 2020). To reduce contingent noise and consider only molecules of bacterial origin, all masses detected in control samples (sterile incubations) and procedural blanks were removed as potential contaminants. Further, we only considered masses detected in all biological replicates at a specific time point for each strain. To check for potential artificial trends being introduced due to this strict filtering threshold, we performed a non-metric multidimensional scaling (NMDS) for both filtered and unfiltered datasets using the Bray-Curtis similarity index. T_0_ samples were not considered as there was no strain-specific release of exometabolites at the time of inoculation. To confirm the statistical reliability of any patterns observed in NMDS plots, cluster analysis was performed using Ward’s linkage (ward.D2) and based on Bray-Curtis distances. Prior to NMDS analysis, the signal intensities of the considered masses in both datasets were normalized. For normalization, the minimum intensity value of the entire processed dataset was multiplied by 5. For each sample, the sum of all intensities higher than this (min*5) value was calculated, and each peak intensity was divided by the sum of the respective sample. For the unfiltered dataset, normalization was performed after removing all singlets, i.e., masses detected only in one sample. All statistical analyses were performed using the software RStudio version 4.1.1. To visualize shared and unique masses at each time point, we created Venn diagrams using both filtered and unfiltered datasets.

Molecular formulae (MF) consisting of C, H, O, N, S and/or P were assigned as described by Koch and Dittmar (2006) to molecular masses with a minimum signal-to-noise ratio of 5. In case of multiple assignments, we did not consider MF with three or more heteroatoms and filtered the remaining in the following order of decreasing priority: NSP, N4P, N4S, N2S and N2P. All MF assigned to FT-ICR-MS detected masses were scanned against the genome-based metabolite prediction list for *P. inhibens*, generated with BioCyc and KEGG databases (Caspi et al., 2016). The functions and ecological relevance of putatively identified exometabolites were analyzed by searching previous lab-based studies as well as environmental datasets (Romano et al., 2014; Osterholz et al., 2015, 2016; Johnson et al., 2016; Wienhausen et al., 2017; Noriega-Ortega et al., 2019).

## Results and Discussion

### Influence of Regulation on Growth Rate, Substrate Utilization and Dissolved Organic Carbon Release

Strain *tdaE*^–^ reached OD_max_ of 1.7 after 42 h, while WT and *pgaR*^–^ grew to OD_max_ of 1.3 and 1.4 after 32 and 60 h, respectively (Figure 1). Growth rate of *tdaE*^–^ (0.22 ± 0.015 h^–1^) was ∼1.5-fold higher than that of the WT (0.15 ± 0.018 h^–1^) while the growth rate of *pgaR*^–^ (0.06 ± 0.008 h^–1^) was ∼3.6-fold and 2.5-fold lower than that of *tdaE*^–^ and WT, respectively, and it took much longer to reach stationary phase. Thus, growth behavior of the WT was different from that of the two mutants, already indicating different impact of the mutations on the physiology.

Differential growth behavior of WT and TDA-negative mutants has been previously investigated and attributed to the high metabolic burden of TDA biosynthesis borne by the WT, while in the mutants this energy burden is absent (Trautwein et al., 2016; Will et al., 2017). TDA targets the cell membrane by collapsing the proton motive force and it has been proposed that *P. inhibens* can resist TDA by counteracting the TDA-induced proton influx with proton efflux mediated by the γ-glutamyl cycle (Wilson et al., 2016). Thus, TDA accumulation in a growing culture may force the WT to allocate a greater share of substrate to energy generation, not only due to TDA biosynthesis, but also due to the proposed proton-efflux self-defense mechanism resulting in faster depletion of glucose compared to the mutants. The higher growth rate of *tdaE*^–^ might therefore also reflect that this mutant does not have to expend additional energy in maintaining the proton gradient. In contrast to *tdaE*^–^, *pgaR*^–^ does not profit from reduced TDA production caused indirectly by absence of the AHL receptor. The absence of both TDA and AHL-based regulation in *pgaR*^–^ may result in higher metabolic costs of unregulated production and release of molecules, as reflected in the high release of DOC and THDAA (Supplementary Table 1).

Growth of the bacterial strains and consumption of glucose was reflected in decreasing DOC concentrations in the culture media. Excluding the remaining glucose from the DOC concentrations at T_3_ revealed that the organic compounds released by *tdaE*^–^, WT and *pgaR*^–^ made up 534 μmol L^–1^, 903 μmol L^–1^ and 1,735 μmol L^–1^ DOC, respectively. The higher and potentially unregulated release of organic compounds in absence of AHL- and TDA-mediated signaling emphasizes the crucial importance of both regulatory mechanisms for cellular functioning.

### Differential Exometabolome Composition of Wild Type and Mutants

**FIGURE 2:**
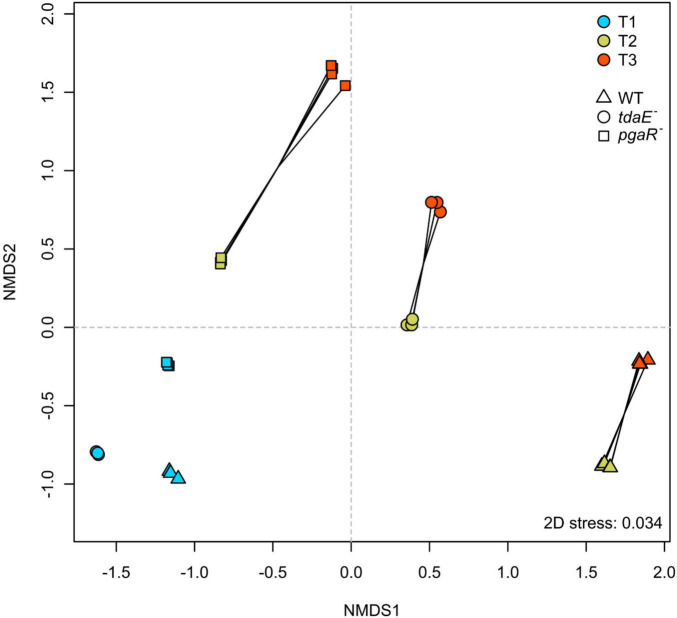
Similarity among the exometabolome samples of *P. inhibens* DSM 17395 wild type (WT), *tdaE*^**–**^ mutant and *pgaR*^**–**^ mutant over time. Non-metric multidimensional scaling (NMDS) was performed for the filtered dataset using Bray-Curtis similarity index. Stress value for the plot is 0.034. All biological replicates of each strain at each sampling point are shown. Color code indicates sampling time points; T_1_ in blue, T_2_ in green and T_3_ in red. Symbols represent the strains; triangle for WT, square for *pgaR*^**–**^ and circle for *tdaE*^**–**^.

The unfiltered dataset obtained from ESI-negative FT-ICR-MS analysis consisted of 17,755 masses ranging from 92 to 1,000 Dalton, of which 996 masses met our stringent criteria of being detected in all biological replicates. Majority of the masses did, however, not meet this criterion, e.g., were present in only 3 out of 4 replicates owing to the natural variability among biological replicates. NMDS analysis of the normalized datasets revealed similar clustering patterns for both filtered and unfiltered datasets (Figure 2 and Supplementary Figure 2), confirming that the filtering thresholds did not introduce artificial trends. Thus, the filtered dataset is highly robust, reproducible and only contains molecules that are definitely of bacterial origin and consistently present at the specific growth phases of the respective strain. Stress value of 0.03 for the NMDS of the filtered dataset (0.08 for unfiltered; Supplementary Figure 2) indicates that it is a good representation of the calculated distance matrix and thus of the similarity among the samples (Figure 2). Samples collected during the lag (T_1_), mid-exponential (T_2_) and early stationary (T_3_) phases were clearly separated from each other for each strain. T_1_ samples of all three strains clustered closely, while T_2_ and T_3_ samples of each strain diverged from T_1_ samples of the respective strain. Thus, the difference in exometabolome composition of each strain with time is consistent with the differential physiological state of bacteria at different growth phases (Noriega-Ortega et al., 2019). T_2_ and T_3_ samples of the WT, *pgaR*^–^ and *tdaE*^–^ were distinctly separated from each other with the highest divergence observed between WT and *pgaR*^–^ (Figure 2). Thus, all three strains released a differential suite of compounds or the same compounds at differential rates, and exometabolome composition developed most differently between WT and *pgaR*^–^. Dendrograms confirmed that the samples clustered consistently with the NMDS groups and the divergences described above were statistically significant (Supplementary Figure 3).

**FIGURE 3:**
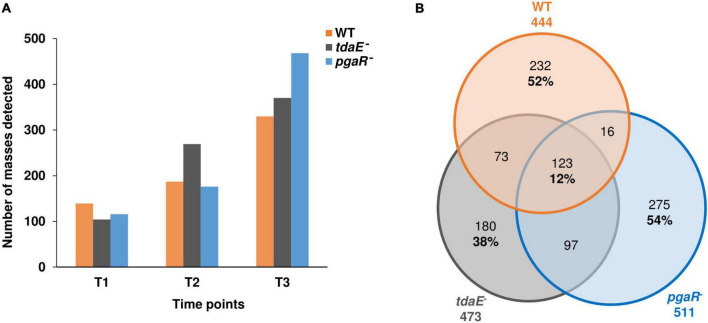
Overall richness of exometabolomes. **(A)** Number of masses detected in the exometabolome of *P. inhibens* DSM 17395 wild type (WT), *tdaE*^**–**^ mutant and *pgaR*^**–**^ mutant at lag (T_1_), mid-exponential (T_2_) and early stationary (T_3_) growth phases. **(B)** Venn diagram showing the number of unique and shared masses detected in the exometabolome of *P. inhibens* DSM 17395 WT (orange), *tdaE*^**–**^ mutant (gray) and *pgaR*^**–**^ mutant (blue) over all time points. Only masses detected in all biological replicates of each strain were considered.

The number of detected masses increased from lag (T_1_) to exponential phase (T_2_) and was highest at early stationary phase (T_3_), prior to cell lysis (Figure 3A). Consistent with the higher release of DOC and THDAA observed for *pgaR*^–^, this mutant also exhibited the highest number of detected masses at T_3_ (463). Considering all growth phases, we detected a total of 444, 473 and 511 masses in the exometabolomes of the WT, *tdaE*^–^ and *pgaR*^–^, respectively. Of these, 232 (52%), 180 (38%) and 275 (54%) masses were strain-specific for the WT, *tdaE*^–^ and *pgaR*^–^, respectively, while only 123 of all detected masses (12%) were shared by all three strains and could be considered as a “core exometabolome,” not affected by QS and/or TDA production (Figure 3B). At exponential and stationary growth phases, 40–60% of the masses detected in the exometabolome of any strain were unique to that strain and only 10% were shared masses (Supplementary Figure 4). We are confident that the lack of overlap observed is not a result of exclusion of masses due to the filtering thresholds, as for the unfiltered dataset, 80–90% of masses detected for each strain were unique to that strain and only 2% of the masses were found in all three exometabolomes (Supplementary Figure 5). In terms of Bray-Curtis dissimilarity, the exometabolomes of *tdaE*^–^ and *pgaR*^–^ were ∼60 and 90% dissimilar, respectively, to that of the WT and ∼70% dissimilar to each other (Supplementary Figure 6).

From statistical analysis of the relative abundance of detected masses, there is comprehensive support for our hypothesis that the exometabolomes of the mutants are substantially different from that of the WT. Further, compared to the exometabolome of *pgaR*^–^, the exometabolome of *tdaE*^–^ was more similar to that of the WT. TdaE catalyzes the first step unique to TDA biosynthesis using the universal tropone precursor and the pathway before and after this step involves several tropone intermediates (Brock et al., 2014b; Wang et al., 2016; Duan et al., 2020). Inactivation of the gene encoding TdaE may result in accumulation of tropolones synthesized prior to TDA synthesis. Different tropolones were shown to act as autoregulatory signaling molecules, for example in antagonistic symbiosis of *Burkholderia plantarii* with rice plants (Duan et al., 2020). This could also be the case for *P. inhibens* where the function of tropone derivatives remains unknown (Thiel et al., 2010; Duan et al., 2020). Thus, presence of the various tropolones along with a functional AHL-based QS may result in *tdaE*^–^ having an exometabolome more similar to the WT. However, since TDA as a signal molecule is part of a global regulatory system and can influence the expression of ∼10% of the total genes, many of which are involved in interactions, its absence may explain the exometabolome of *tdaE*^–^ being ∼60% dissimilar to that of WT. Both AHL and TDA bind to the LuxR receptor to function as signaling molecules and the absence of this receptor in *pgaR*^–^ renders this mutant incapable of AHL- and TDA-based global regulation, resulting in a significantly distinct exometabolome.

High chemodiversity of exometabolomes of single strains has been shown previously where the exometabolome composition also varied depending on physiological cues, growth phase and substrate utilized by the strains (Rosselló-Mora et al., 2008; Romano et al., 2014; Fiore et al., 2015; Johnson et al., 2016; Noriega-Ortega et al., 2019). The lifestyle of *P. inhibens* as a host-associated bacterium and biofilm former along with its elaborate secondary metabolism (Brinkhoff et al., 2004; Martens et al., 2007; Thiel et al., 2010; Seyedsayamdost et al., 2011; Thole et al., 2012) suggests that most of the exometabolites, such as siderophores, TDA, roseobacticide family of algicides, and other bioactive tropone derivatives and volatiles are linked to cellular metabolism (Brock et al., 2014a; Duan et al., 2021) and released to serve as currencies for species interactions rather than arising from errors in metabolic processes or due to overflow metabolism. However, the disruption of TDA production and QS, potentially resulting in the absence of many tropone-derived secondary metabolites as well as a global regulatory system, significantly altered the chemical composition of the exometabolomes.

### Molecular Formulae Assignment and Putative Annotation of Exometabolites

Of the 996 masses in the filtered dataset, unique molecular formulae (MF) could be assigned to 603 masses. A higher percentage of MF was assigned to masses detected in T_1_ samples (75%) while ∼62% of masses detected in T_2_ and T_3_ samples could be assigned MF. The proportion of nitrogen-containing MF increased from 47% at T_1_ to 77% at T_3_ for *pgaR*^–^ and from 38% (T_1_) to 49% (T_3_) for *tdaE*^–^, while it decreased from 56% to 23% for the WT. The WT is known to deplete external ammonium in early exponential phase to rapidly build up nitrogen-containing intracellular metabolites and biopolymers (DNA, RNA, proteins), and high cellular nitrogen levels coincide with the production of TDA (Trautwein et al., 2018). However, absence of TDA and global regulation in the mutants may result in more nitrogen-containing exometabolites being released, as is consistent with the THDAA data (Supplementary Tables 1, 2).

The proportion of sulfur-containing MF increased only for the WT from 11% at T_1_ to 37% at T_3_. *Phaeobacter* spp. are known to produce a range of sulfur containing metabolites such as TDA, tropone, tropolone and its derivates as well as several volatiles involved in species interactions (Thiel et al., 2010; Brock et al., 2014a,b). Although volatile organic compounds could not be detected by FT-ICR-MS in this study, several other sulfur containing compounds produced as intermediates of the TDA biosynthesis pathway may be released for the WT. Most of these intermediates are generated after the TdaE catalysis step (Duan et al., 2020), likely reflecting the lower percentage of sulfur-containing MF detected for *tdaE*^–^. Moreover, reduction of sulfate for incorporation into organic molecules is an energy intensive process. Some coastal bacterioplankton communities preferentially process organic sulfur, assimilating organic matter with a 1.6-fold bias toward sulfur-containing molecules (Vorobev et al., 2018). Thus, obtaining pre-reduced sulfur from organosulfur compounds released by other microbes, could be a survival strategy for many bacterial taxa (Moran and Durham, 2019). This may be of particular importance in biofilms, the major environmental niche of *P. inhibens*, where cooperative behavior, metabolite exchange and cross-feeding are required (Xavier and Foster, 2007).

For annotation of exometabolites, we scanned all MF against the genome-predicted metabolite list for *P. inhibens* generated with BioCyc and KEGG databases, resulting in the putative assignment of 36, 21 and 26 exometabolites for *tdaE*^–^, *pgaR*^–^ and WT, respectively (Supplementary Table 3). Thus, most of the MF detected in the exometabolomes were not predicted by the genome and/or could not be matched to any metabolite database. Some of the exometabolites could be a result of paralogous and overflow metabolism; however, there might also be wrong or incomplete annotations in the database reducing the annotation efficiency. Our putative annotation is supported by the detection/annotation of several identical metabolites in other studies with similar approaches (Romano et al., 2014; Fiore et al., 2015; Johnson et al., 2016; Wienhausen et al., 2017) as indicated in Supplementary Table 3. We found, as expected, the major QS molecule *N*-3-hydroxydecanoyl-homoserine lactone in the exometabolome of all three strains, and TDA present in the WT exometabolome and absent from that of the *tdaE*^–^. We suspected that *pgaR*^–^ might produce TDA toward late exponential phase since it has all the biosynthesis genes and TDA is known to auto induce its own biosynthesis in some strains (Geng and Belas, 2010; Berger et al., 2012). However, TDA was not detected, confirming that its production was highly down-regulated in this mutant (Berger et al., 2011; Beyersmann et al., 2017). Relative abundance of TDA showed a log_2_ fold change of −1.35 from T_2_ to T_3_ for the WT. Relative abundance of AHL showed a log_2_ fold change of −2.38, −1.96 and +0.69 for the WT, *tdaE*^–^ and *pgaR*^–^, respectively, from T_1_ to T_2_. From T_2_ to T_3_, log_2_ fold change of AHL was −0.33, −1.17 and −0.64 for the WT, *tdaE*^–^ and *pgaR*^–^, respectively (Supplementary Table 4).

### Differential Detection of Amino Acids in the Exometabolomes

Concentrations of THDAA measured by HPLC increased during exponential and stationary growth phases of all strains, reaching a final amino acid concentration of 31.3 μM, 44.4 μM and 96.8 μM for WT, *tdaE*^–^ and *pgaR*^–^, respectively. During exponential and stationary phases of all three strains, glutamate, glycine and alanine dominated the pool, constituting together nearly 60 mol% of THDAA (Supplementary Table 2), as observed previously (Wienhausen et al., 2017). Concentrations of DFAA remained below the detection limit of HPLC analysis for all three strains; however, masses matching tryptophan, tyrosine, phenylalanine and histidine were detected in the exometabolomes by FT-ICR-MS. Most MF identical to amino acids and biosynthetic precursors and derivatives of amino acids were also putatively detected in the DOM of North Sea mesocosms and Mediterranean Sea water samples (Osterholz et al., 2015; Martínez-Pérez et al., 2017). However, we cannot exclude that these masses reflect other compounds with equal elemental composition. Both HPLC and FT-ICR-MS data indicate that the WT releases less amino acids than the mutants (Supplementary Tables 1, 3), pointing to the differential regulation of primary and secondary metabolism in the mutants (Yan et al., 2021).

From the FT-ICR-MS data, phenylalanine was detected in all exometabolomes except for stationary phase of the WT. In *P. inhibens*, tropone, TDA and roseobacticides are all biosynthesized in a QS-regulated manner from phenylacetyl-CoA or phenylpyruvate, which are mainly produced by degradation of phenylalanine (Thiel et al., 2010; Seyedsayamdost et al., 2011; Berger et al., 2012; Brock et al., 2014a; Rabe et al., 2014; Wang et al., 2016). Since QS is switched on at higher cell densities attained in late exponential phase, in the WT phenylalanine is redirected toward biosynthesis of tropone and its derivatives, explaining its absence in the exometabolome at stationary phase. Compared to the WT, relative abundance of phenylalanine was 6-fold and 3-fold higher in exponential phase cultures of *pgaR*^–^ and *tdaE*^–^, respectively. Transcriptome analysis and labeling experiments showed that phenylalanine is not catabolized for energy in the WT, but only used as precursor for TDA production (Will, 2018), which could explain why we detected the amino acid in exometabolome of both mutants where TDA production is absent. The production of tropone and its derivatives generated from phenylacetyl-CoA depends on a dedicated metabolic pathway including TdaE and multiple steps of this pathway are QS-regulated (Brock et al., 2014a,b; Wang et al., 2016; Duan et al., 2020). Thus, the absence of global regulation in the mutants may reduce conversion of phenylalanine into tropone products, resulting in release of the amino acid.

### Ecologically Relevant Metabolites Putatively Identified From Molecular Formulae

**FIGURE 4:**
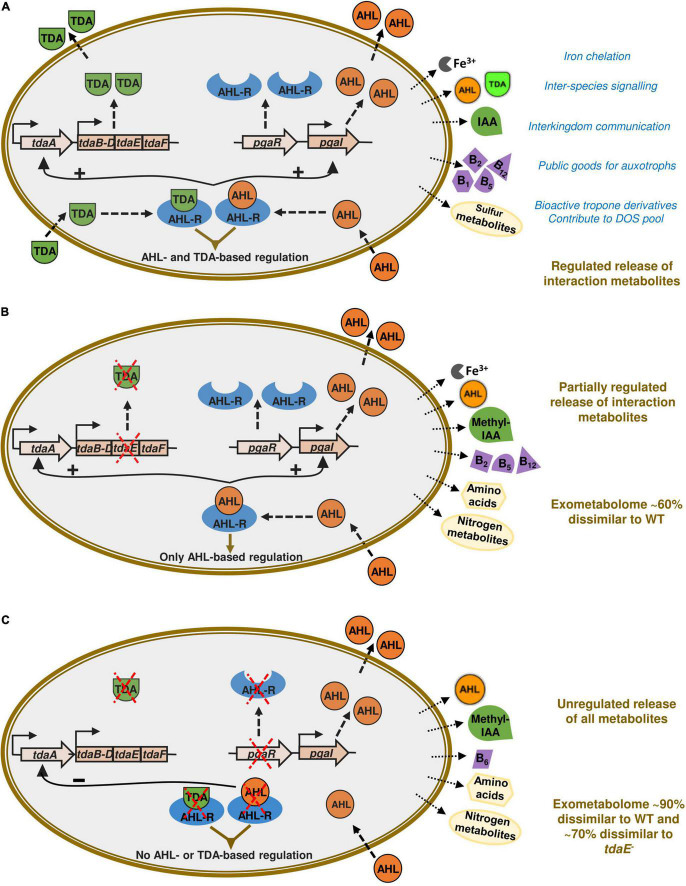
Impact of TDA and AHL signaling on exometabolome of *P. inhibens* DSM 17395. The *pgaR* gene is encoding for the *N*-acyl-homoserine lactone regulator (AHL-R) and *pgaI* for the AHL synthase. The *tdaA* gene encodes the transcriptional regulator for the tropodithietic acid (TDA) biosynthesis gene cluster, *tdaB-F*. The observed impact of TDA and AHL-mediated global regulation on the exometabolome based on differential detection of ecologically relevant metabolites is indicated by arrows outside the cells. In the wild type **(A)**, AHL and TDA molecules interact with the AHL regulator to regulate global gene expression. This signaling also results in upregulation of TDA and AHL biosynthesis genes. In pure cultures of the *tdaE*^**–**^ mutant **(B)**, there is an intact AHL-based regulation, but no TDA production due to a non-functional *tdaE* gene. Therefore, TDA-based regulation is absent resulting in the absence of certain interaction metabolites such as vitamins and auxins. In the *pgaR*^**–**^ mutant **(C)**, there is neither AHL- nor TDA-based regulation since there is no regulator for the molecules to bind to. This results in the unregulated release of nitrogen containing metabolites but many exometabolites vital for species interactions are absent. This figure is based on QS signaling model proposed by Beyersmann et al. (2017). The original figure is licensed under Creative Commons Attribution 4.0 International License (https://creativecommons.org/licenses/by/4.0/) thereby permitting modifications.

The putatively annotated compounds included late biosynthetic precursors of and/or vitamins B_1_, B_2_, B_5_, B_6_, B_7_ and B_12_, amino acids and biosynthetic precursors and derivatives of amino acids, the phytohormone indole acetic acid (IAA) and its methylated form, siderophore components, AHLs and TDA (Figure 4). Many of these compounds were detected only for specific strains and/or at specific growth phases (Table 1 and Supplementary Table 3).

**TABLE 1:** Putatively annotated ecologically relevant metabolites detected differentially in the exometabolomes of *P. inhibens* DSM 17395 WT and the mutants *pgaR*^**–**^ and *tdaE*^**–**^.

Molecular formula	Putative metabolites	Function	WT			*pgaR* ^–^			*tdaE* ^–^		
			T1	T2	T3	T1	T2	T3	T1	T2	T3
C_14_H_25_NO_4_	*N*-3-hydroxydecanoyl-L-homoserine lactone	Quorum sensing	**+**	**+**	**+**	**+**	**+**	**+**	**+**	**+**	**+**
C_8_H_4_O_3_S_2_	Tropodithietic acid	Antimicrobial, signaling	**–**	**+**	**+**	**–**	**–**	**–**	**–**	**–**	**–**
C_10_H_9_NO_2_	Indole acetic acid (IAA)	Auxin	**–**	**–**	**+**	**–**	**–**	**–**	**–**	**–**	**–**
C_11_H_11_NO_2_	Methyl (indole-3-yl) acetate	IAA related	**–**	**–**	**–**	**–**	**–**	**+**	**–**	**–**	**+**
C_11_H_12_N_2_O_2_	Tryptophan	Amino acid IAA precursor	**–**	**–**	**–**	**–**	**–**	**+**	**–**	**–**	**–**
C_9_H_11_NO_2_	Phenylalanine	Amino acid (precursor of tropone, TDA, algicide)	**+**	**+**	**–**	**+**	**+**	**+**	**+**	**+**	**+**
C_9_H_11_NO_3_	Tyrosine	Amino acid	**–**	**–**	**–**	**–**	**–**	**+**	**–**	**–**	**–**
C_6_H_9_N_3_O_2_	Histidine	Amino acid	**–**	**–**	**–**	**–**	**–**	**–**	**+**	**+**	**–**
C_10_H_15_N_2_O_8_P	Thiamine phosphate	Vitamin B1	**–**	**–**	**+**	**–**	**–**	**–**	**–**	**–**	**–**
C_17_H_20_N_4_O_6_	Riboflavin	Vitamin B2	**–**	**–**	**+**	**–**	**–**	**–**	**–**	**–**	**+**
C_9_H_17_NO_5_	Pantothenate	Vitamin B5	**–**	**–**	**+**	**–**	**–**	**–**	**–**	**–**	**+**
C_8_H_9_NO_3_	Pyridoxal	Vitamers of B6	**–**	**–**	**–**	**–**	**–**	**+**	**–**	**–**	**–**
C_8_H_11_NO_3_	Pyridoxine		**–**	**–**	**–**	**–**	**–**	**+**	**–**	**–**	**–**
C_8_H_12_N_2_O_2_	Pyridoxamine		**–**	**–**	**–**	**–**	**–**	**+**	**–**	**–**	**–**
C_10_H_18_N_2_O_3_	Dethiobiotin	Vitamin B7 precursor	**+**	**+**	**+**	**+**	**+**	**+**	**+**	**+**	**+**
C_14_H_18_N_2_O_4_	Alpha-ribazole	Vitamin B12 precursor	**–**	**+**	**+**	**–**	**–**	**–**	**–**	**+**	**+**
C_14_H_19_N_2_O_7_P	Alpha-ribazole-5-phosphate	Vitamin B12 precursor	**+**	**–**	**–**	**–**	**–**	**–**	**–**	**–**	**–**
C_7_H_6_O_4_	3,4-dihydroxy benzoate (3,4-DHB)	Siderophore building block	**–**	**+**	**+**	**–**	**–**	**–**	**–**	**+**	**+**

*+/– indicates presence/absence of metabolites, and T_1_ (lag), T_2_ (mid-exponential), T_3_ (early stationary) indicate the growth phases in which the metabolites were detected.*

We detected thiamine monophosphate (vitamin B_1_) only in the exometabolome of WT at T_3_. Riboflavin (vitamin B_2_) and pantothenate (vitamin B_5_) were detected in the exometabolomes of *tdaE*^–^ and WT at T_3_. Alpha-ribazole, a biosynthetic precursor of cobalamin (vitamin B_12_) was detected in the exometabolomes of WT and *tdaE*^–^ at T_2_ and T_3_. Pyridoxal, the dephosphorylated form of vitamin B_6_ and alpha-ribazole were also putatively detected in DOM of a mesocosm experiment simulating North Sea phytoplankton bloom (Osterholz et al., 2015) and Mediterranean Sea water samples (Martínez-Pérez et al., 2017). Vitamins and their precursors are well-known factors orchestrating phytoplankton-bacteria interactions since ∼50% of > 300 phytoplankton species are auxotrophic for vitamin B_12_, ∼25% for vitamin B_1_, ∼8% for vitamin B_7_, and > 60% of marine bacterial species are unable to synthesize vitamin B_12_ (Croft et al., 2005; Sañudo-Wilhelmy et al., 2006, 2014; Grant et al., 2014; Cruz-López and Maske, 2016; Cruz-López et al., 2018; Cooper et al., 2019). Exchange or one-way supply of vitamins is already known for some roseobacters such as *Sulfitobacter*, *Dinoroseobacter* and *Ruegeria* spp. (Wagner-Döbler et al., 2010; Durham et al., 2015; Cruz-López and Maske, 2016; Cruz-López et al., 2018; Cooper et al., 2019). *P. inhibens* being a biofilm former and often found associated with phytoplankton in mesocosm studies and natural phytoplankton blooms (González et al., 2000; Grossart et al., 2005; Rao et al., 2006; Thole et al., 2012; Green et al., 2015; Majzoub et al., 2019), may be a key player in vitamin-based species interactions. Based on the differential detection of various vitamins and their precursors in the exometabolomes, we hypothesize that in *P. inhibens* export of vitamin B_1_ is influenced by AHL- and TDA-based regulation while export of alpha-ribazole, the vitamin B_12_ precursor, and vitamins B_2_ and B_5_ is influenced only by AHL-mediated regulation (Table 1). Our findings provide a novel insight into the influence of signaling molecules on the release of vitamins and/or their precursors into the marine environment (Figure 4).

Microbial interdependencies extend beyond vitamins to signaling molecules and growth-promoting auxins. We detected tryptophan only in the exometabolome of *pgaR*^–^ at T_3_, and IAA only in the exometabolome of the WT at T_3_ (Table 1). Both tryptophan and IAA have been identified as key metabolites in the interaction between *P. inhibens* and the coccolithophore *Emiliana huxleyi* (Segev et al., 2016) as well as another roseobacter, *Sulfitobacter* sp. SA11 and the diatom *Pseudo-nitzschia multiseries* (Amin et al., 2015). *P. inhibens* produces IAA as a growth promoting/inhibiting molecule for *E. huxleyi* from endogenous tryptophan but can also shunt exogenous tryptophan (from its host) primarily toward IAA production. Detection of IAA only in the WT exometabolome suggests that its biosynthesis from endogenous tryptophan and its release may require both AHL- and TDA-based regulation. In the exometabolome of the TDA-negative mutant, neither IAA not tryptophan was detected, suggesting the channeling of tryptophan toward other metabolic pathways, as the strain still possesses functional AHL-based QS. In the absence of both regulatory mechanisms, tryptophan is released. Thus, we suspect that TDA-based regulation plays a major role in directing tryptophan toward IAA biosynthesis.

Comparing our data with naturally-derived DOM from a mesocosm experiment simulating a North Sea phytoplankton bloom (Osterholz et al., 2015) and a North Sea transect study (Osterholz et al., 2016) revealed 208 and 67 matches, respectively, for masses with unique molecular formulae assigned. 95% of the matches from the mesocosm study were present not only in the mesocosm samples but also in the sea water samples (Supplementary Table 3), indicating that at least on the molecular formula level, a large fraction of the detected metabolites in our study are indeed part of the natural DOM. These matches include certain universal exometabolites such as amino acids and their precursors but also metabolites that are widespread among roseobacters such as quorum sensing molecules (Ziesche et al., 2015). Some metabolites like vitamins and their precursors are more valuable in the open ocean as auxotrophy is widely distributed (Sañudo-Wilhelmy et al., 2014) and even among prototrophs, only some release the vitamins for common use. *Phaeobacter* spp. have been isolated from various marine habitats such as coastal waters, harbor surfaces, microbial biofilms and in association with eukaryotes including diatoms and coccolithophores (Seyedsayamdost et al., 2011; Thole et al., 2012; Gram et al., 2015; Majzoub et al., 2019). A recent study revealed their widespread natural occurrence in the marine pelagial along a longitudinal Pacific transect (Freese et al., 2017). Although the contribution of individual bacterial exometabolomes to marine DOM remains largely unknown (Bercovici et al., 2022) and abundance of *Phaeobacter* spp. in metagenomic databases from the oceans is very low, their potential for host-associated and biofilm-forming lifestyle renders it ecologically significant to study the exometabolites released by them into the DOM.

Differential production of exometabolites by the *P. inhibens* strains investigated in this study reveals the ecological advantage conferred to the WT by AHL- and TDA-based signaling, despite the metabolic burden (Figure 4). Furthermore, these two regulatory mechanisms seem to be interlinked in influencing production and release of amino acids and vitamins as well as their precursors, signaling molecules, siderophores and other secondary metabolites, relevant for organismal interactions. In the absence of these regulatory mechanisms, the bacterial exometabolome is markedly different, indicating high relevance of these mechanisms in structured environments such as biofilms and phycospheres where interdependencies are vital.

## Conclusion

*Phaeobacter inhibens* was often found associated with marine eukaryotes, and its ability to form biofilms, accompanied with production of secondary metabolites like TDA enables the bacterium to even invade pre-established biofilms (Rao et al., 2006). In microbial consortia, release of high-value metabolites such as vitamins, amino acids, auxins and siderophores is indispensable for the collective community success. The non-targeted characterization of the diverse exometabolomes of WT and mutants of *P. inhibens* by ultrahigh-resolution FT-ICR-MS revealed that AHL- and TDA-based QS are important factors regulating production and release of a multitude of compounds. Metabolites of known ecological relevance were differentially detected in the exometabolomes of mutants and WT. Absence of AHL- and TDA-based global regulatory systems resulted in the absence of several of these key exometabolites and in a substantially different exometabolome composition. Thus, we conclude that in our model organism the full suite of compounds released is highly regulated by at least two core processes—AHL-based QS and TDA production. The fact that the exometabolomes of the individual strains contain more unique (40–60%) than shared (10–12%) exometabolites stands testimony to our conclusion. Although TDA production is limited to a few genera, the impact of TDA on exometabolome composition could serve as a model for other secondary metabolites, possibly also having regulatory function. Moreover, AHL-based QS being widespread in *Proteobacteria* might also have a strong influence on exometabolomes of many other bacteria. Hence, this study underlines the significance of global regulatory mechanisms for production of complex and diverse exometabolomes, critical for bacteria dwelling in biofilm consortia and host-associations.

## Data Availability Statement

The raw data supporting the conclusions of this article will be made available by the authors, without undue reservation.

## Author Contributions

SS performed the experimental laboratory work, data analysis, data interpretation, and manuscript drafting. MB assisted in genetic manipulation experiments and manuscript revision. TB and JN advised throughout the course of research, contributed to data interpretation, and manuscript revision. All authors contributed to designing of the research and also contributed to manuscript finalization.

## Conflict of Interest

The authors declare that the research was conducted in the absence of any commercial or financial relationships that could be construed as a potential conflict of interest.

## Publisher’s Note

All claims expressed in this article are solely those of the authors and do not necessarily represent those of their affiliated organizations, or those of the publisher, the editors and the reviewers. Any product that may be evaluated in this article, or claim that may be made by its manufacturer, is not guaranteed or endorsed by the publisher.

## References

[B1] Amin S. A. Hmelo L. R. van Tol H. M. Durham B. P. Carlson L. T. Heal K. R. (2015). Interaction and signalling between a cosmopolitan phytoplankton and associated bacteria. *Nature* 522 98–101. 10.1038/nature14488 26017307

[B2] An J. H. Goo E. Kim H. Seo Y.-S. Hwang I. (2014). Bacterial quorum sensing and metabolic slowing in a cooperative population. *Proc. Natl. Acad. Sci. U S A.* 111 14912–14917. 10.1073/pnas.1412431111 25267613 PMC4205598

[B3] Antonova E. S. Hammer B. K. (2011). Quorum-sensing autoinducer molecules produced by members of a multispecies biofilm promote horizontal gene transfer to *Vibrio cholerae*. *FEMS Microbiol. Lett.* 322 68–76. 10.1111/j.1574-6968.2011.02328.x 21658103

[B4] Atkinson S. Williams P. (2009). Quorum sensing and social networking in the microbial world. *J. R. Soc. Interface* 6 959–978. 10.1098/rsif.2009.0203 19674996 PMC2827448

[B5] Barofsky A. Vidoudez C. Pohnert G. (2009). Metabolic profiling reveals growth stage variability in diatom exudates. *Limnol. Oceanogr. Methods* 7 382–390. 10.4319/lom.2009.7.382

[B6] Becker J. Berube P. Follett C. Waterbury J. Chisholm S. DeLong E. (2014). Closely related phytoplankton species produce similar suite of dissolved organic matter. *Front. Microbiol.* 5:111. 10.3389/fmicb.2014.00111 24748874 PMC3975126

[B7] Bercovici S. K. Dittmar T. Niggemann J. (2022). The detection of bacterial exometabolites in marine dissolved organic matter through ultrahigh-resolution mass spectrometry. *Limnol. Oceanogr. Methods.* 3, 1–11. 10.1002/lom3.10491

[B8] Berger M. Brock N. L. Liesegang H. Dogs M. Preuth I. Simon M. (2012). Genetic analysis of the upper phenylacetate catabolic pathway in the production of tropodithietic acid by *Phaeobacter gallaeciensis*. *Appl. Environ. Microbiol.* 78 3539–3551. 10.1128/AEM.07657-7611 22407685 PMC3346364

[B9] Berger M. Neumann A. Schulz S. Simon M. Brinkhoff T. (2011). Tropodithietic acid production in *Phaeobacter gallaeciensis* is regulated by N-acyl homoserine lactone-mediated quorum sensing. *J. Bacteriol.* 193 6576–6585. 10.1128/JB.05818-5811 21949069 PMC3232910

[B10] Beyersmann P. G. Tomasch J. Son K. Stocker R. Göker M. Wagner-Döbler I. (2017). Dual function of tropodithietic acid as antibiotic and signaling molecule in global gene regulation of the probiotic bacterium *Phaeobacter inhibens*. *Sci. Rep.* 7:730. 10.1038/s41598-017-00784-787 28389641 PMC5429656

[B11] Blokesch M. (2012). A quorum sensing-mediated switch contributes to natural transformation of *Vibrio cholerae*. *Mob. Genet. Elements* 2 224–227. 10.4161/mge.22284 23446800 PMC3575429

[B12] Brinkhoff T. Giebel H.-A. Simon M. (2008). Diversity, ecology, and genomics of the roseobacter clade: a short overview. *Arch. Microbiol.* 189 531–539. 10.1007/s00203-008-0353-y 18253713

[B13] Brinkhoff T. Bach G. Heidorn T. Liang L. Schlingloff A. Simon M. (2004). Antibiotic production by a roseobacter clade-affiliated species from the German Wadden Sea and its antagonistic effects on indigenous isolates. *Appl. Environ. Microbiol.* 70 2560–2565. 10.1128/AEM.70.4.2560-2565.2003 15066861 PMC383154

[B14] Brock N. L. Menke M. Klapschinski T. A. Dickschat J. S. (2014a). Marine bacteria from the Roseobacter clade produce sulfur volatiles *via* amino acid and dimethylsulfoniopropionate catabolism. *Org. Biomol. Chem.* 12 4318–4323. 10.1039/C4OB00719K 24848489

[B15] Brock N. L. Nicolay A. Dickschat J. S. (2014b). Biosynthesis of the antibiotic tropodithietic acid by the marine bacterium *Phaeobacter inhibens*. *Chem. Commun.* 50 5487–5489. 10.1039/C4CC01924E 24723119

[B16] Buchan A. González J. M. Moran M. A. (2005). Overview of the marine Roseobacter lineage. *Appl. Environ. Microbiol.* 71 5665–5677. 10.1128/AEM.71.10.5665-5677.2005 16204474 PMC1265941

[B17] Buchan A. LeCleir G. R. Gulvik C. A. González J. M. (2014). Master recyclers: features and functions of bacteria associated with phytoplankton blooms. *Nat. Rev. Microbiol.* 12 686–698. 10.1038/nrmicro3326 25134618

[B18] Caspi R. Billington R. Ferrer L. Foerster H. Fulcher C. A. Keseler I. M. (2016). The MetaCyc database of metabolic pathways and enzymes and the BioCyc collection of pathway/genome databases. *Nucleic Acids Res.* 44 D471–D480. 10.1093/nar/gkv1164 26527732 PMC4702838

[B19] Cooper M. B. Kazamia E. Helliwell K. E. Kudahl U. J. Sayer A. Wheeler G. L. (2019). Cross-exchange of B-vitamins underpins a mutualistic interaction between *Ostreococcus tauri* and *Dinoroseobacter shibae*. *ISME J.* 13 334–345. 10.1038/s41396-018-0274-y 30228381 PMC6331578

[B20] Croft M. T. Lawrence A. D. Raux-Deery E. Warren M. J. Smith A. G. (2005). Algae acquire vitamin B12 through a symbiotic relationship with bacteria. *Nature* 438 90–93. 10.1038/nature04056 16267554

[B21] Cruz-López R. Maske H. (2016). The vitamin B1 and B12 required by the marine dinoflagellate *Lingulodinium polyedrum* can be provided by its associated bacterial community in culture. *Front. Microbiol.* 7:560. 10.3389/fmicb.2016.00560 27199906 PMC4858720

[B22] Cruz-López R. Maske H. Yarimizu K. Holland N. A. (2018). The B-vitamin mutualism between the dinoflagellate *Lingulodinium polyedrum* and the bacterium Dinoroseobacter shibae. *Front. Mar. Sci.* 5:274. 10.3389/fmars.2018.00274

[B23] Cude W. N. Mooney J. Tavanaei A. A. Hadden M. K. Frank A. M. Gulvik C. A. (2012). Production of the antimicrobial secondary metabolite indigoidine contributes to competitive surface colonization by the marine Roseobacter Phaeobacter sp. *Appl. Environ. Microbiol.* 78 4771–4780. 10.1128/AEM.00297-212 22582055 PMC3416362

[B24] Cude W. Buchan A. (2013). Acyl-homoserine lactone-based quorum sensing in the Roseobacter clade: complex cell-to-cell communication controls multiple physiologies. *Front. Microbiol.* 4:336. 10.3389/fmicb.2013.00336 24273537 PMC3824088

[B25] Dower W. J. Miller J. F. Ragsdale C. W. (1988). High efficiency transformation of *E. coli* by high voltage electroporation. *Nucleic Acids Res.* 16 6127–6145. 10.1093/nar/16.13.6127 3041370 PMC336852

[B26] Duan Y. Petzold M. Saleem-Batcha R. Teufel R. (2020). Bacterial tropone natural products and derivatives: overview of their biosynthesis, bioactivities, ecological role and biotechnological potential. *ChemBioChem* 21 2384–2407. 10.1002/cbic.201900786 32239689 PMC7497051

[B27] Duan Y. Toplak M. Hou A. Brock N. L. Dickschat J. S. Teufel R. (2021). A flavoprotein dioxygenase steers bacterial tropone biosynthesis i coenzyme A-ester oxygenolysis and ring epoxidation. *J. Am. Chem. Soc.* 143 10413–10421. 10.1021/jacs.1c04996 34196542 PMC8283759

[B28] Durham B. P. Sharma S. Luo H. Smith C. B. Amin S. A. Bender S. J. (2015). Cryptic carbon and sulfur cycling between surface ocean plankton. *Proc. Natl. Acad. Sci. U S A.* 112 453–457. 10.1073/pnas.1413137112 25548163 PMC4299198

[B29] Fiore C. L. Longnecker K. Kido Soule M. C. Kujawinski E. B. (2015). Release of ecologically relevant metabolites by the cyanobacterium *Synechococcus elongatus* CCMP 1631. *Environ. Microbiol.* 17 3949–3963. 10.1111/1462-2920.12899 25970745

[B30] Freese H. M. Methner A. Overmann J. (2017). Adaptation of surface-associated bacteria to the open ocean: a genomically distinct subpopulation of Phaeobacter gallaeciensis colonizes Pacific mesozooplankton. *Front. Microbiol.* 8:1659. 10.3389/fmicb.2017.01659 28912769 PMC5583230

[B31] Gao M. Zheng H. Ren Y. Lou R. Wu F. Yu W. (2016). A crucial role for spatial distribution in bacterial quorum sensing. *Sci. Rep.* 6:34695. 10.1038/srep34695 27698391 PMC5048177

[B32] Geng H. Belas R. (2010). Expression of tropodithietic acid biosynthesis is controlled by a novel autoinducer. *J. Bacteriol.* 192 4377–4387. 10.1128/JB.00410-410 20601479 PMC2937372

[B33] Gillard J. Frenkel J. Devos V. Sabbe K. Paul C. Rempt M. (2013). Metabolomics enables the structure elucidation of a diatom sex pheromone. *Angew. Chemie Int. Ed.* 52 854–857. 10.1002/anie.201208175 23315901

[B34] González J. M. Simó R. Massana R. Covert J. S. Casamayor E. O. Pedrós-Alió C. (2000). Bacterial community structure associated with a dimethylsulfoniopropionate-producing North Atlantic algal bloom. *Appl. Environ. Microbiol.* 66 4237–4246. 10.1128/AEM.66.10.4237-4246.2000 11010865 PMC92291

[B35] Gram L. Grossart H.-P. Schlingloff A. Kiørboe T. (2002). Possible quorum sensing in marine snow bacteria: production of acylated homoserine lactones by Roseobacter strains isolated from marine snow. *Appl. Environ. Microbiol.* 68 4111–4116. 10.1128/AEM.68.8.4111-4116.2002 12147515 PMC123997

[B36] Gram L. Rasmussen B. B. Wemheuer B. Bernbom N. Ng Y. Y. Porsby C. H. (2015). Phaeobacter inhibens from the Roseobacter clade has an environmental niche as a surface colonizer in harbors. *Syst. Appl. Microbiol.* 38 483–493. 10.1016/j.syapm.2015.07.006 26343311

[B37] Grant M. A. A. Kazamia E. Cicuta P. Smith A. G. (2014). Direct exchange of vitamin B12 is demonstrated by modelling the growth dynamics of algal-bacterial cocultures. *ISME J.* 8 1418–1427. 10.1038/ismej.2014.9 24522262 PMC4069406

[B38] Green D. H. Echavarri-Bravo V. Brennan D. Hart M. C. (2015). Bacterial diversity associated with the coccolithophorid algae *Emiliania huxleyi* and *Coccolithus pelagicus* f. braarudii. *Biomed Res. Int.* 2015:194540. 10.1155/2015/194540 26273594 PMC4529885

[B39] Grossart H.-P. Levold F. Allgaier M. Simon M. Brinkhoff T. (2005). Marine diatom species harbour distinct bacterial communities. *Environ. Microbiol.* 7 860–873. 10.1111/j.1462-2920.2005.00759.x 15892705

[B40] Hahnke S. Brock N. L. Zell C. Simon M. Dickschat J. S. Brinkhoff T. (2013). Physiological diversity of Roseobacter clade bacteria co-occurring during a phytoplankton bloom in the North Sea. *Syst. Appl. Microbiol.* 36 39–48. 10.1016/j.syapm.2012.09.004 23265193

[B41] Hom E. F. Y. Murray A. W. (2014). Niche engineering demonstrates a latent capacity for fungal-algal mutualism. *Science* 345 94–98. 10.1126/science.1253320 24994654 PMC4409001

[B42] Johnson W. M. Kido Soule M. C. Kujawinski E. B. (2016). Evidence for quorum sensing and differential metabolite production by a marine bacterium in response to DMSP. *ISME J.* 10 2304–2316. 10.1038/ismej.2016.6 26882264 PMC4989321

[B43] Kell D. B. Brown M. Davey H. M. Dunn W. B. Spasic I. Oliver S. G. (2005). Metabolic footprinting and systems biology: the medium is the message. *Nat. Rev. Microbiol.* 3 557–565. 10.1038/nrmicro1177 15953932

[B44] Koch B. P. Dittmar T. (2006). From mass to structure: an aromaticity index for high-resolution mass data of natural organic matter. *Rapid Commun. Mass Spectrom.* 20, 926–932. 10.1002/rcm.2386

[B45] Kujawinski E. B. Longnecker K. Blough N. V. Vecchio R. Del, Finlay L. (2009). Identification of possible source markers in marine dissolved organic matter using ultrahigh resolution mass spectrometry. *Geochim. Cosmochim. Acta* 73 4384–4399. 10.1016/j.gca.2009.04.033

[B46] Landa M. Burns A. S. Roth S. J. Moran M. A. (2017). Bacterial transcriptome remodeling during sequential co-culture with a marine dinoflagellate and diatom. *ISME J.* 11 2677–2690. 10.1038/ismej.2017.117 28731474 PMC5702724

[B47] Latifi A. Winson M. K. Foglino M. Bycroft B. W. Stewart G. S. A. B. Lazdunski A. (1995). Multiple homologues of LuxR and LuxI control expression of virulence determinants and secondary metabolites through quorum sensing in *Pseudomonas aeruginosa* PAO1. *Mol. Microbiol.* 17 333–343. 10.1111/j.1365-2958.1995.mmi_17020333.x 7494482

[B48] Lawrence D. Fiegna F. Behrends V. Bundy J. G. Phillimore A. B. Bell T. (2012). Species interactions alter evolutionary responses to a novel environment. *PLoS Biol.* 10:e1001330. 10.1371/journal.pbio.1001330 22615541 PMC3352820

[B49] Liang L. (2003). *Investigation of Secondary Metabolites of North Sea Bacteria: Fermentation, Isolation, Structure Elucidation and Bioactivity.* Germany: Georg-August-Universität.

[B50] Longnecker K. Futrelle J. Coburn E. Kido Soule M. C. Kujawinski E. B. (2015). Environmental metabolomics: databases and tools for data analysis. *Mar. Chem.* 177 366–373. 10.1016/j.marchem.2015.06.012

[B51] Lunau M. Lemke A. Dellwig O. Simon M. (2006). Physical and biogeochemical controls of microaggregate dynamics in a tidally affected coastal ecosystem. *Limnol. Oceanogr.* 51 847–859. 10.4319/lo.2006.51.2.0847

[B52] Luo H. Moran M. A. (2014). Evolutionary ecology of the marine Roseobacter clade. *Microbiol. Mol. Biol. Rev.* 78 573–587. 10.1128/MMBR.00020-14 25428935 PMC4248658

[B53] Majzoub M. E. Beyersmann P. G. Simon M. Thomas T. Brinkhoff T. Egan S. (2019). Phaeobacter inhibens controls bacterial community assembly on a marine diatom. *FEMS Microbiol. Ecol.* 95:fiz060. 10.1093/femsec/fiz060 31034047

[B54] Mansson M. Gram L. Larsen T. O. (2011). Production of bioactive secondary metabolites by marine Vibrionaceae. *Mar. Drugs* 9 1440–1468. 10.3390/md9091440 22131950 PMC3225927

[B55] Martens T. Gram L. Grossart H.-P. Kessler D. Müller R. Simon M. (2007). Bacteria of the Roseobacter clade show potential for secondary metabolite production. *Microb. Ecol.* 54 31–42. 10.1007/s00248-006-9165-9162 17351813

[B56] Martínez-Pérez A. M. Nieto-Cid M. Osterholz H. Catalá T. S. Reche I. Dittmar T. (2017). Linking optical and molecular signatures of dissolved organic matter in the Mediterranean Sea. *Sci. Rep.* 7:3436. 10.1038/s41598-017-03735-3734 28611434 PMC5469803

[B57] McRose D. L. Baars O. Seyedsayamdost M. R. Morel F. M. M. (2018). Quorum sensing and iron regulate a two-for-one siderophore gene cluster in *Vibrio harveyi*. *Proc. Natl. Acad. Sci. U S A.* 115 7581–7586. 10.1073/pnas.1805791115 29954861 PMC6055174

[B58] Merder J. Freund J. A. Feudel U. Hansen C. T. Hawkes J. A. Jacob B. (2020). ICBM-OCEAN: processing ultrahigh-resolution mass spectrometry data of complex molecular mixtures. *Anal. Chem.* 92 6832–6838. 10.1021/acs.analchem.9b05659 32298576

[B59] Miller M. B. Bassler B. L. (2001). Quorum sensing in bacteria. *Annu. Rev. Microbiol.* 55 165–199. 10.1146/annurev.micro.55.1.165 11544353

[B60] Moran M. A. Durham B. P. (2019). Sulfur metabolites in the pelagic ocean. *Nat. Rev. Microbiol.* 17 665–678. 10.1038/s41579-019-0250-251 31485034

[B61] Noriega-Ortega B. E. Wienhausen G. Mentges A. Dittmar T. Simon M. Niggemann J. (2019). Does the chemodiversity of bacterial exometabolomes sustain the chemodiversity of marine dissolved organic matter? *Front. Microbiol.* 10:215. 10.3389/fmicb.2019.00215 30837961 PMC6382689

[B62] Osterholz H. Dittmar T. Niggemann J. (2014). Molecular evidence for rapid dissolved organic matter turnover in Arctic fjords. *Mar. Chem.* 160 1–10. 10.1016/j.marchem.2014.01.002

[B63] Osterholz H. Niggemann J. Giebel H.-A. Simon M. Dittmar T. (2015). Inefficient microbial production of refractory dissolved organic matter in the ocean. *Nat. Commun.* 6:7422. 10.1038/ncomms8422 26084883

[B64] Osterholz H. Singer G. Wemheuer B. Daniel R. Simon M. Niggemann J. (2016). Deciphering associations between dissolved organic molecules and bacterial communities in a pelagic marine system. *ISME J.* 10 1717–1730. 10.1038/ismej.2015.231 26800236 PMC4918438

[B65] Paczia N. Nilgen A. Lehmann T. Gätgens J. Wiechert W. Noack S. (2012). Extensive exometabolome analysis reveals extended overflow metabolism in various microorganisms. *Microb. Cell Fact.* 11:122. 10.1186/1475-2859-11-122 22963408 PMC3526501

[B66] Patterson A. G. Jackson S. A. Taylor C. Evans G. B. Salmond G. P. C. Przybilski R. (2016). Quorum sensing controls adaptive immunity through the regulation of multiple CRISPR-Cas systems. *Mol. Cell* 64 1102–1108. 10.1016/j.molcel.2016.11.012 27867010 PMC5179492

[B67] Patzelt D. Wang H. Buchholz I. Rohde M. Gröbe L. Pradella S. (2013). You are what you talk: quorum sensing induces individual morphologies and cell division modes in Dinoroseobacter shibae. *ISME J.* 7 2274–2286. 10.1038/ismej.2013.107 23823498 PMC3834844

[B68] Piekarski T. Buchholz I. Drepper T. Schobert M. Wagner-Doebler I. Tielen P. (2009). Genetic tools for the investigation of Roseobacter clade bacteria. *BMC Microbiol.* 9:265. 10.1186/1471-2180-9-265 20021642 PMC2811117

[B69] Pohlner M. Dlugosch L. Wemheuer B. Mills H. Engelen B. Reese B. K. (2019). The majority of active Rhodobacteraceae in marine sediments belong to uncultured genera: a molecular approach to link their distribution to environmental conditions. *Front. Microbiol.* 10:659. 10.3389/fmicb.2019.00659 31001232 PMC6454203

[B70] Porsby C. H. Webber M. A. Nielsen K. F. Piddock L. J. V. Gram L. (2011). Resistance and tolerance to tropodithietic acid, an antimicrobial in aquaculture, is hard to select. *Antimicrob. Agents Chemother.* 55 1332–1337. 10.1128/AAC.01222-1210 21263047 PMC3067165

[B71] Prol García M. J. D’Alvise P. W. Rygaard A. M. Gram L. (2014). Biofilm formation is not a prerequisite for production of the antibacterial compound tropodithietic acid in *Phaeobacter inhibens* DSM17395. *J. Appl. Microbiol.* 117 1592–1600. 10.1111/jam.12659 25284322

[B72] Rabe P. Klapschinski T. A. Brock N. L. Citron C. A. D’Alvise P. Gram L. (2014). Synthesis and bioactivity of analogues of the marine antibiotic tropodithietic acid. *Beilstein J. Org. Chem.* 10 1796–1801. 10.3762/bjoc.10.188 25161739 PMC4142847

[B73] Rao D. Webb J. S. Kjelleberg S. (2006). Microbial colonization and competition on the marine alga Ulva australis. *Appl. Environ. Microbiol.* 72 5547–5555. 10.1128/AEM.00449-446 16885308 PMC1538698

[B74] Romano S. Dittmar T. Bondarev V. Weber R. J. M. Viant M. R. Schulz-Vogt H. N. (2014). Exo-metabolome of *Pseudovibrio sp*. FO-BEG1 analyzed by ultra-high resolution mass spectrometry and the effect of phosphate limitation. *PLoS One* 9:e96038. 10.1371/journal.pone.0096038 24787987 PMC4008564

[B75] Rosselló-Mora R. Lucio M. Peña A. Brito-Echeverría J. López-López A. Valens-Vadell M. (2008). Metabolic evidence for biogeographic isolation of the extremophilic bacterium *Salinibacter ruber*. *ISME J.* 2 242–253. 10.1038/ismej.2007.93 18239610

[B76] Sañudo-Wilhelmy S. A. Gobler C. J. Okbamichael M. Taylor G. T. (2006). Regulation of phytoplankton dynamics by vitamin B12. *Geophys. Res. Lett.* 33:L04604. 10.1029/2005GL025046

[B77] Sañudo-Wilhelmy S. A. Gómez-Consarnau L. Suffridge C. Webb E. A. (2014). The role of B Vitamins in marine biogeochemistry. *Ann. Rev. Mar. Sci.* 6 339–367. 10.1146/annurev-marine-120710-100912 24050603

[B78] Segev E. Wyche T. P. Kim K. H. Petersen J. Ellebrandt C. Vlamakis H. (2016). Dynamic metabolic exchange governs a marine algal-bacterial interaction. *eLife* 5:e17473. 10.7554/eLife.17473 27855786 PMC5148602

[B79] Seyedsayamdost M. R. Case R. J. Kolter R. Clardy J. (2011). The jekyll-and-hyde chemistry of *Phaeobacter gallaeciensis*. *Nat. Chem.* 3 331–335. 10.1038/nchem.1002 21430694 PMC3376411

[B80] Thiel V. Brinkhoff T. Dickschat J. S. Wickel S. Grunenberg J. Wagner-Döbler I. (2010). Identification and biosynthesis of tropone derivatives and sulfur volatiles produced by bacteria of the marine Roseobacter clade. *Org. Biomol. Chem.* 8 234–246. 10.1039/B909133E 20024154

[B81] Thole S. Kalhoefer D. Voget S. Berger M. Engelhardt T. Liesegang H. (2012). Phaeobacter gallaeciensis genomes from globally opposite locations reveal high similarity of adaptation to surface life. *ISME J.* 6 2229–2244. 10.1038/ismej.2012.62 22717884 PMC3504968

[B82] Thoma S. Schobert M. (2009). An improved *Escherichia coli* donor strain for diparental mating. *FEMS Microbiol. Lett.* 294 127–132. 10.1111/j.1574-6968.2009.01556.x 19431232

[B83] Trautwein K. Hensler M. Wiegmann K. Skorubskaya E. Wöhlbrand L. Wünsch D. (2018). The marine bacterium *Phaeobacter inhibens* secures external ammonium by rapid buildup of intracellular nitrogen stocks. *FEMS Microbiol. Ecol.* 94:fiy154. 10.1093/femsec/fiy154 30124819 PMC6122490

[B84] Trautwein K. Will S. E. Hulsch R. Maschmann U. Wiegmann K. Hensler M. (2016). Native plasmids restrict growth of *Phaeobacter inhibens* DSM 17395: energetic costs of plasmids assessed by quantitative physiological analyses. *Environ. Microbiol.* 18 4817–4829. 10.1111/1462-2920.13381 27233797

[B85] Urvoy M. Labry C. L’Helguen S. Lami R. (2022). Quorum sensing regulates bacterial processes that play a major role in marine biogeochemical cyles. *Front. Mar. Sci.* 9:834337. 10.3389/fmars.2022.834337

[B86] Villas-Bôas S. G. Noel S. Lane G. A. Attwood G. Cookson A. (2006). Extracellular metabolomics: a metabolic footprinting approach to assess fiber degradation in complex media. *Anal. Biochem.* 349 297–305. 10.1016/j.ab.2005.11.019 16356465

[B87] Vorobev A. Sharma S. Yu M. Lee J. Washington B. J. Whitman W. B. (2018). Identifying labile DOM components in a coastal ocean through depleted bacterial transcripts and chemical signals. *Environ. Microbiol.* 20 3012–3030. 10.1111/1462-2920.14344 29968336

[B88] Wagner-Döbler I. Biebl H. (2006). Environmental biology of the marine Roseobacter lineage. *Annu. Rev. Microbiol.* 60 255–280. 10.1146/annurev.micro.60.080805.142115 16719716

[B89] Wagner-Döbler I. Ballhausen B. Berger M. Brinkhoff T. Buchholz I. Bunk B. (2010). The complete genome sequence of the algal symbiont *Dinoroseobacter shibae*: a hitchhiker’s guide to life in the sea. *ISME J.* 4 61–77. 10.1038/ismej.2009.94 19741735

[B90] Walker L. R. Tfaily M. M. Shaw J. B. Hess N. J. Paša-Tolić L. Koppenaal D. W. (2017). Unambiguous identification and discovery of bacterial siderophores by direct injection 21 tesla fourier transform ion cyclotron resonance mass spectrometry. *Metallomics* 9 82–92. 10.1039/c6mt00201c 27905613

[B91] Wang R. Gallant E. Seyedsayamdost M. R. (2016). Investigation of the genetics and biochemistry of roseobacticide production in the Roseobacter clade bacterium *Phaeobacter inhibens*. *mBio* 7:e02118–15. 10.1128/mBio.02118-2115 27006458 PMC4807370

[B92] Wheeler G. L. Tait K. Taylor A. Brownlee C. Joint I. (2006). Acyl-homoserine lactones modulate the settlement rate of zoospores of the marine alga Ulva intestinalis *via* a novel chemokinetic mechanism. *Plant. Cell Environ.* 29 608–618. 10.1111/j.1365-3040.2005.01440.x 17080611

[B93] Wichard T. Poulet S. A. Halsband-Lenk C. Albaina A. Harris R. Liu D. (2005). Survey of the chemical defence potential of diatoms: screening of fifty species for α,β,γ,δ-unsaturated aldehydes. *J. Chem. Ecol.* 31 949–958. 10.1007/s10886-005-3615-z 16124261

[B94] Wienhausen G. Noriega-Ortega B. E. Niggemann J. Dittmar T. Simon M. (2017). The exometabolome of two model strains of the Roseobacter group: a marketplace of microbial metabolites. *Front. Microbiol.* 8:1985. 10.3389/fmicb.2017.01985 29075248 PMC5643483

[B95] Will S. E. (2018). *Experimental and Theoretical Analyses of the Metabolic Role of the 262-kb Plasmid in the Context of the Endogenously Produced Antibiotic Tropodithietic Acid in Phaeobacter Inhibens DSM 17395.* Germany: Technische Universität Braunschweig.

[B96] Will S. E. Neumann-Schaal M. Heydorn R. L. Bartling P. Petersen J. Schomburg D. (2017). The limits to growth - energetic burden of the endogenous antibiotic tropodithietic acid in Phaeobacter inhibens DSM 17395. *PLoS One* 12:e0177295. 10.1371/journal.pone.0177295 28481933 PMC5421792

[B97] Wilson M. Z. Wang R. Gitai Z. Seyedsayamdost M. R. (2016). Mode of action and resistance studies unveil new roles for tropodithietic acid as an anticancer agent and the γ-glutamyl cycle as a proton sink. *Proc. Natl. Acad. Sci. U S A.* 113 1630–1635. 10.1073/pnas.1518034113 26802120 PMC4760781

[B98] Xavier J. B. Foster K. R. (2007). Cooperation and conflict in microbial biofilms. *Proc. Natl. Acad. Sci. U S A.* 104 876–881. 10.1073/pnas.0607651104 17210916 PMC1783407

[B99] Yan C. Li X. Zhang G. Zhu Y. Bi J. Hao H. (2021). Quorum sensing-mediated and growth phase-dependent tegulation of metabolic pathways in *Hafnia alvei* H4. *Front. Microbiol.* 12:567942. 10.3389/fmicb.2021.567942 33737914 PMC7960787

[B100] Zan J. Choi O. Meharena H. Uhlson C. L. Churchill M. E. A. Hill R. T. (2015). A solo luxI-type gene directs acylhomoserine lactone synthesis and contributes to motility control in the marine sponge symbiont *Ruegeria sp*. KLH11. *Microbiology* 161 50–56. 10.1099/mic.0.083956-0 25355937 PMC4811643

[B101] Zan J. Cicirelli E. M. Mohamed N. M. Sibhatu H. Kroll S. Choi O. (2012). A complex LuxR-LuxI type quorum sensing network in a roseobacterial marine sponge symbiont activates flagellar motility and inhibits biofilm formation. *Mol. Microbiol.* 85 916–933. 10.1111/j.1365-2958.2012.08149.x 22742196 PMC3429658

[B102] Zan J. Liu Y. Fuqua C. Hill R. T. (2014). Acyl-homoserine lactone quorum sensing in the Roseobacter clade. *Int. J. Mol. Sci.* 15 654–669. 10.3390/ijms15010654 24402124 PMC3907830

[B103] Zech H. Thole S. Schreiber K. Kalhöfer D. Voget S. Brinkhoff T. (2009). Growth phase-dependent global protein and metabolite profiles of Phaeobacter gallaeciensis strain DSM 17395, a member of the marine Roseobacter clade. *Proteomics* 9 3677–3697. 10.1002/pmic.200900120 19639587

[B104] Zhu L. Chen T. Xu L. Zhou Z. Feng W. Liu Y. (2020). Effect and mechanism of quorum sensing on horizontal transfer of multidrug plasmid RP4 in BAC biofilm. *Sci. Total Environ.* 698:134236. 10.1016/j.scitotenv.2019.134236 31493577

[B105] Ziesche L. Bruns H. Dogs M. Wolter L. Mann F. Wagner-Döbler I. (2015). Homoserine lactones, methyl oligohydroxybutyrates, and other extracellular metabolites of macroalgae-associated bacteria of the roseobacter clade: identification and functions. *ChemBioChem* 16 2094–2107. 10.1002/cbic.201500189 26212108

