# Supplementary material for: Impact of Quorum Sensing and Tropodithietic Acid Production on the Exometabolome of Phaeobacter inhibens
Source: Front Microbiol. 2022 Jun 21;13:917969. doi: 10.3389/fmicb.2022.917969 (PMC9253639; doi:10.3389/fmicb.2022.917969)
Supplement: Supplementary file 1 [file Data_Sheet_1.pdf]

Supplementary material to "Impact of quorum sensing and tropodithietic acid production on the exometabolome of *Phaeobacter inhibens*"

Supplementary Methods

Transformation of *E. coli*

A 5 mL overnight culture of *E. coli* ST18 was grown in half strength MB (MB_{50}) supplemented with 50 µg/mL 5-aminolevulinic acid (ALA; Sigma-Aldrich, Munich, Germany) in a 15 mL sterile culture tube at 37°C and 100 rpm. It was then diluted 50-fold into 175 mL of MB_{50}+ALA in a 1 L Erlenmeyer flask and grown to an OD_{600} of ~0.6 (logarithmic growth), cooled down in ice water with gentle swirling for 15 minutes and then centrifuged (10 min, 10,000 x g, 4°C). The cell pellet obtained was washed thrice with 50 mL of 10% glycerol and after the last washing step, re-suspended in 0.4 mL of 10% glycerol, distributed in 40 µL aliquots, flash-frozen in liquid nitrogen and stored at -80°C until further use. For transformation, 40 µL of the cells was mixed with 1 µL of the ligation mixture and transferred to cooled electroporation cuvette (0.2 cm gap). The mixture was electroporated at 2.5 V for 5 ms, immediately returned to ice, supplemented with 1 mL MB_{50}+ALA and transferred to a 10 mL sterile culture tube. The cells were cultured at 37°C and 120 rpm for 2 h and then plated on MB_{50} medium supplemented with 50 µg/mL ALA and 50 µg/mL kanamycin (Km_{50}) for selection of transformants.

Conjugation of *P. inhibens* WP14 and *E. coli* ST18

The donor strain, ST18 transformed with pBBR1MCS2-*tdaE* plasmid, was grown to an OD_{600} of ~0.6 (37°C, 100 rpm, ~6 h) in MB_{50}+ALA. The recipient strain, *P. inhibens*

WP14, was grown to an OD_{600} of ~3 (28°C, 100 rpm, ~24 h) in MB_{50}. For conjugation, liquid cultures were mixed in 5:1 and 10:1 donor to recipient ratios, centrifuged (5 min, 9,000 x g), supernatant discarded, the pellet re-suspended and transferred onto a completely dry MB_{50}+ALA plate, which was then incubated at 28°C. After ~24 h, the pellet was scraped from the plate, resuspended in PBS buffer, serially diluted and plated on MB_{50}+Km_{50} plates. Passaging of single colonies was repeated thrice in MB_{50} supplemented with 50 µg/mL of kanamycin and 30 µg/mL of gentamycin to obtain only transconjugants without non-conjugated recipient and residual donor cells. The transconjugants were confirmed by PCR amplification, and restoration of the wild type phenotype was assessed with an antimicrobial bioassay and measurement of pigmentation.

Supplementary Figure and Table Legends

Figure S1: Antimicrobial activity and pigmentation of *P. inhibens* DSM 17395 wild type (WT), *tdaE*$^-$ mutant and the complemented transconjugant. **(A)** Zone of inhibition against *Pseudoalteromonas tunicata* determined by agar diffusion assays using cell-free culture fluids extracted from late exponential phase cultures. **(B)** Brown pigmentation of the three strains at late exponential phase measured by spectroscopy at 398 nm. Error bars depict standard deviation of three biological replicates. Asterisks denote statistically significant differences between WT and *tdaE*$^-$ mutant as well as the complemented transconjugant and *tdaE*$^-$ mutant.

Figure S2: Similarity among the exometabolome samples of *P. inhibens* DSM 17395 wild type (WT), *tdaE*$^-$ mutant and *pgaR*$^-$ mutant over time. Non-metric multidimensional scaling (NMDS) was performed for the unfiltered dataset using Bray-Curtis similarity index. Stress

value for the plot is 0.083. All biological replicates of each strain at each sampling point are shown. Color code indicates sampling time points; T_1 in blue, T_2 in yellow and T_3 in orange. Symbols represent the strains; triangle for WT, square for *pgaR*$^-$ and circle for *tdaE*$^-$.

Figure S3: Dendrograms constructed by performing a cluster analysis of the filtered **(A)** and unfiltered **(B)** FT-ICR-MS datasets using Ward's linkage (ward.D2) and based on Bray-Curtis similarity index. Color code indicates sampling time points; T_1 in blue, T_2 in yellow and T_3 in orange. Symbols represent the strains; triangle for WT, square for *pgaR*$^-$ and circle for *tdaE*$^-$.

Figure S4: Richness of exometabolomes during mid-exponential **(A)** and early stationary **(B)** growth phases. Number of masses detected in the exometabolomes of *P. inhibens* DSM 17395 wildtype (WT), *tdaE*$^-$ mutant and *pgaR*$^-$ mutant are colour coded in orange, grey and blue, respectively. Only masses detected in all biological replicates of each strain were considered. Sampling time points T_2 and T_3 correspond to mid-exponential and early stationary growth phases respectively.

Figure S5: Venn diagram constructed using the unfiltered dataset and showing the number of unique and shared masses detected in the exometabolome of *P. inhibens* DSM 17395 WT (orange), *tdaE*$^-$ mutant (grey) and *pgaR*$^-$ mutant (blue) over all time points.

Figure S6: Heatmap based on Bray-Curtis dissimilarity index with molecular masses and their relative abundance detected in the exometabolomes of *P. inhibens* DSM 17395 wild type (WT), *tdaE*$^-$ mutant (WP14) and *pgaR*$^-$ mutant (WP52). Colour gradient from blue to red indicates increasing dissimilarity.

Table S1: Concentrations of glucose, total hydrolysable dissolved amino acids (THDAA), dissolved organic carbon (DOC), DOC minus glucose and solid-phase extracted DOC (SPE-DOC) for *P. inhibens* DSM 17395 wild type (WT), *tdaE*$^-$ and *pgaR*$^-$ mutants during inoculation (T_0), lag (T_1), mid-exponential (T_2) and early stationary (T_3) growth phases. Values given are mean standard ± deviation.

Table S2: Mol % (± standard deviation) of detected amino acids in total hydrolysable dissolved amino acids (THDAA) in the exometabolome of *P. inhibens* DSM 17395 wild type and the two mutants, *tdaE*$^-$ and *pgaR*$^-$ during different growth phases. Concentration of valine in all samples and of phenylalanine in the T_0 samples could not be determined (nd) by HPLC analysis due to interference from a large unknown peak.

Table S3: Masses detected in exometabolomes of *P. inhibens* DSM 17395 wild type (WT) and the two mutants *tdaE*$^-$ and *pgaR*$^-$ and the molecular formulas (MF) assigned to these masses along with their putative annotation. T_1, T_2 and T_3 correspond to lag, mid-exponential and early stationary growth phases of the bacterial strains. a). m/z values detected by FT-ICR-MS analysis; b). molecular formulas assigned; c) putative annotation using KEGG and BioCyc databases; +, detected; -, not detected.

Table S4: Normalized relative abundance of masses corresponding to tropodithietic acid (TDA) and *N*-3-hydroxydecanoyl-L-homoserine lactone (AHL) at lag (T_1), mid-exponential (T_2) and early stationary (T_3) growth phases of *P. inhibens* DSM 17395 wild type (WT), *tdaE*$^-$ mutant and *pgaR*$^-$ mutant strains. Values are given as mean ± (standard error). TDA was not detected at T_1 for the WT. Relative abundance of TDA showed a $\log_2$ fold change of -1.35 from T_2 to T_3 for the WT. Relative abundance of the AHL showed a $\log_2$ fold change of -2.38, -1.96 and +0.69 for the WT, *tdaE*$^-$ and *pgaR*$^-$, respectively, from T_1 to T_2.

Relative abundance of the AHL showed a log_2 fold change of -0.33, -1.17 and -0.64 for the WT, *tdaE*$^-$ and *pgaR*$^-$, respectively, from T_2 to T_3.

Table S5: Elemental composition and elemental ratios of masses with unique molecular formulas detected over all the sampling time points in the exometabolomes of *P. inhibens* DSM 17395 wild type (WT), *tdaE*$^-$ mutant and *pgaR*$^-$ mutant.

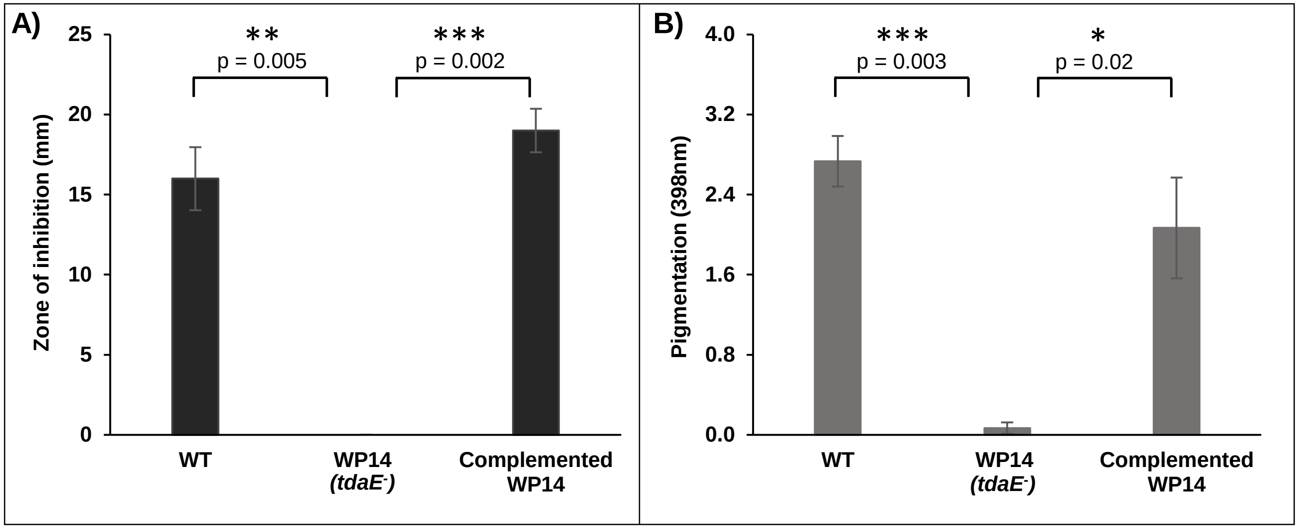

Figure S1: Antimicrobial activity and pigmentation of *P. inhibens* DSM 17395 wild type (WT), *tdaE*⁻ mutant and the complemented transconjugant. **(A)** Zone of inhibition against *Pseudoalteromonas tunicata* determined by agar diffusion assays using cell-free culture fluids extracted from late exponential phase cultures. **(B)** Brown pigmentation of the three strains at late exponential phase measured by spectroscopy at 398 nm. Error bars depict standard deviation of three biological replicates. Asterisks denote statistically significant differences between WT and *tdaE*⁻ mutant as well as the complemented transconjugant and *tdaE*⁻ mutant.

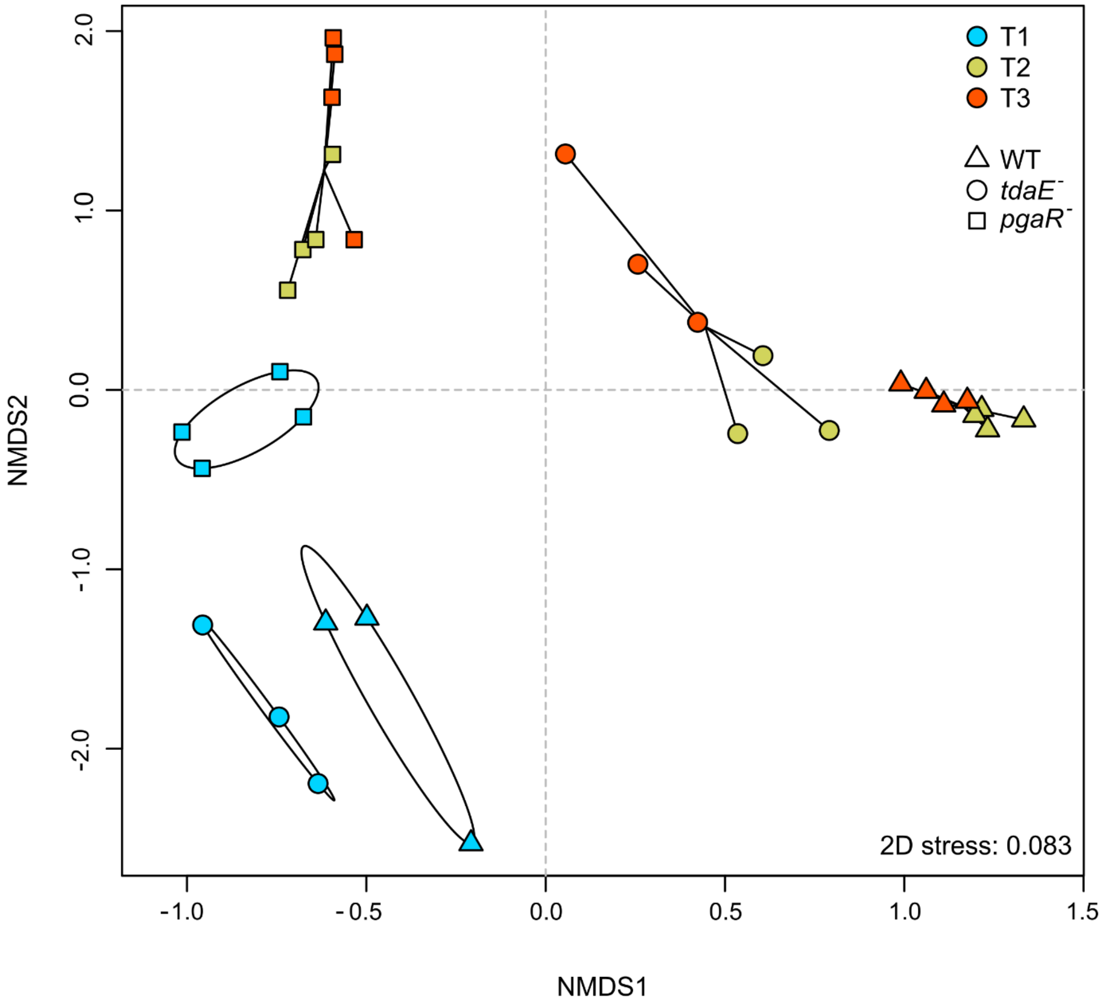

Figure S2: Similarity among the exometabolome samples of *P. inhibens* DSM 17395 wild type (WT), *tdaE*$^{-}$ mutant and *pgaR*$^{-}$ mutant over time. Non-metric multidimensional scaling (NMDS) was performed for the unfiltered dataset using Bray-Curtis similarity index. Stress value for the plot is 0.083. All biological replicates of each strain at each sampling point are shown. Color code indicates sampling time points; T_1 in blue, T_2 in yellow and T_3 in orange. Symbols represent the strains; triangle for WT, square for *pgaR*$^{-}$ and circle for *tdaE*$^{-}$

.

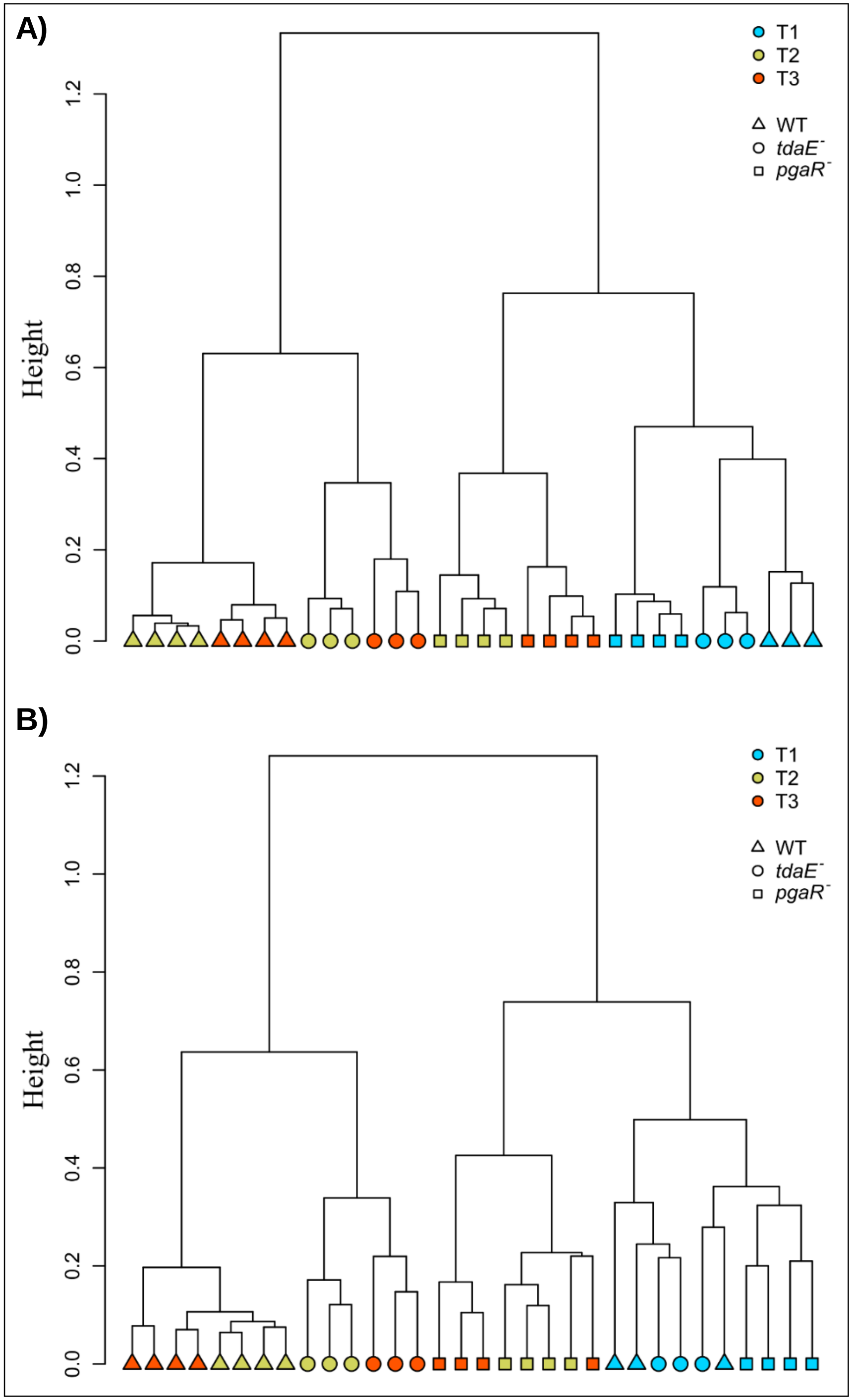

Figure S3: Dendrograms constructed by performing a cluster analysis of the filtered **(A)** and unfiltered **(B)** FT-ICR-MS datasets using Ward's linkage (ward.D2) and based on Bray-Curtis similarity index. Color code indicates sampling time points; T_1 in blue, T_2 in yellow and T_3 in orange. Symbols represent the strains; triangle for WT, square for *pgaR*- and circle for *tdaE*-.

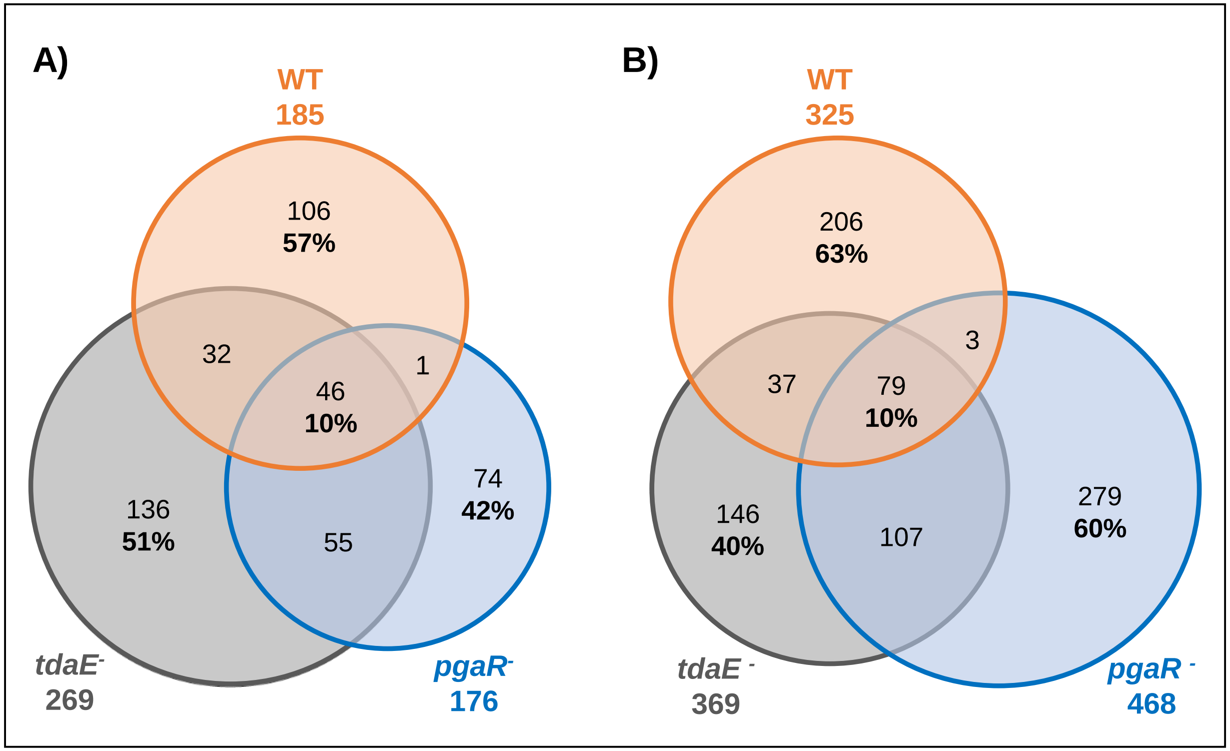

Figure S4: Richness of exometabolomes during mid-exponential **(A)** and early stationary **(B)** growth phases. Number of masses detected in the exometabolomes of *P. inhibens* DSM 17395 wildtype (WT), *tdaE*⁻ and *pgaR*⁻ are colour coded in orange, grey and blue, respectively. Only masses detected in all biological replicates of each strain were considered. Sampling time points T_2 and T_3 correspond to mid-exponential and early stationary growth phases respectively.

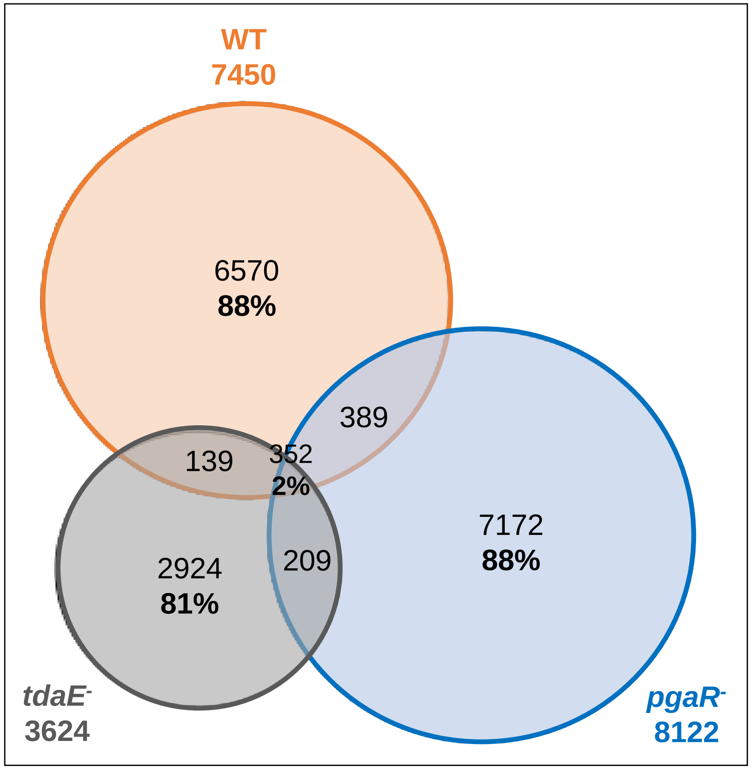

Figure S5: Venn diagram constructed using the unfiltered dataset and showing the number of unique and shared masses detected in the exometabolome of *P. inhibens* DSM 17395 WT (orange), *tdaE*$^{-}$ mutant (grey) and *pgaR*$^{-}$ mutant (blue) over all time points.

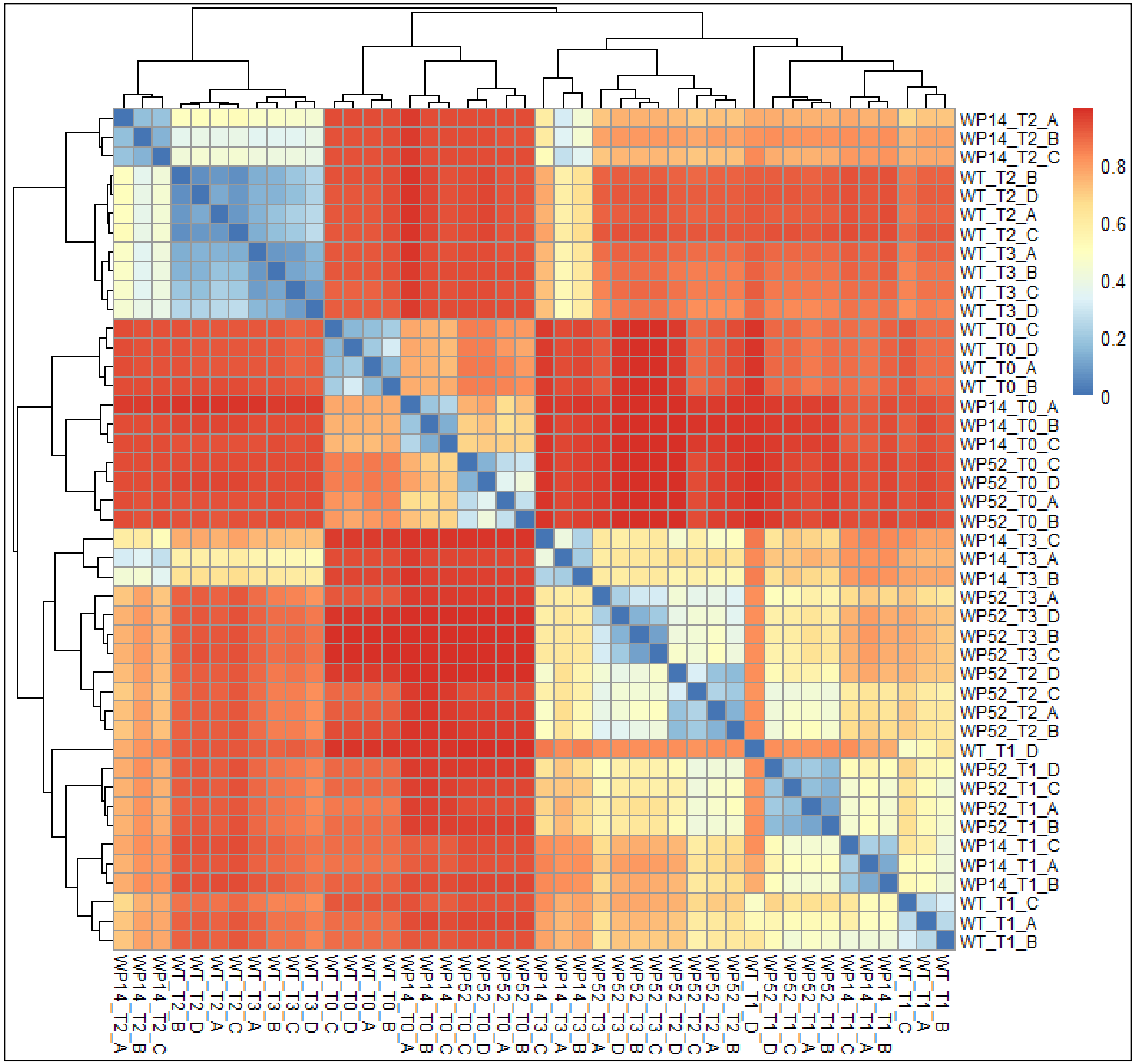

Figure S6: Heatmap based on Bray-Curtis dissimilarity index with molecular masses and their relative abundance detected in the exometabolomes of *P. inhibens* DSM 17395 wild type (WT), *tdaE*[-] mutant (WP14) and *pgaR*[-] mutant (WP52). Colour change from blue to red indicates increasing dissimilarity.

Table S1: Concentrations of glucose, total hydrolysable dissolved amino acids (THDAA), dissolved organic carbon (DOC), DOC minus glucose and solid-phase extracted DOC (SPE-DOC) for *P. inhibens* DSM 17395 wild type (WT), *tdaE*$^-$ and *pgaR*$^-$ mutants during inoculation (T_0), lag (T_1), mid-exponential (T_2) and early stationary (T_3) growth phases. Values given are mean ± standard deviation.

	Time point	*tdaE*$^-$	*pgaR*$^-$	WT
**Glucose (mM)**	T_0	4.77 (±0.02)	4.74 (±0.25)	4.73 (±0.29)
	T_1	4.42 (±0.32)	4.32 (±0.19)	4.29 (±0.21)
	T_2	2.13 (±0.16)	2.37 (±0.29)	2.29 (±0.53)
	T_3	0.004 (±0.00)	0.001 (±0.00)	0.004 (±0.00)
**THDAA (µM)**	T_0	0.06 (±0.00)	0.42 (±0.00)	0.01 (±0.00)
	T_1	0.97 (±0.23)	7.89 (±0.21)	1.59 (±0.39)
	T_2	19.76 (±0.65)	53.66 (±0.03)	12.19 (±0.05)
	T_3	44.41 (±0.09)	96.83 (±0.22)	31.26 (±0.17)
**DOC (mmol/L)**	T_0	23.87 (±0.72)	24.38 (±0.59)	24.84 (±0.21)
	T_1	22.31 (±1.40)	22.40 (±0.52)	22.38 (±0.79)
	T_2	11.33 (±1.60)	13.82 (±0.85)	10.55 (±1.28)
	T_3	0.53 (±0.08)	1.74 (±0.23)	0.91 (±0.07)
**DOC-glucose (mmol/L)**	T_0	-4.754 (±0.30)	-4.059 (±0.09)	-3.539 (±0.69)
	T_1	-4.216 (±1.17)	-3.526 (±0.48)	-3.353 (±0.37)
	T_2	-1.451 (±0.06)	-0.403 (±0.12)	-3.196 (±0.86)
	T_3	0.534 (±0.08)	1.735 (±0.04)	0.903 (±0.04)
**SPE-DOC (µmol/L)**	T_0	52.16 (±13.58)	25.41 (±12.46)	37.53 (±12.81)
	T_1	27.48 (±11.96)	30.00 (±2.88)	41.88 (±14.23)
	T_2	120.03 (±12.71)	201.91 (±3.62)	285.89 (±4.71)
	T_3	206.78 (±4.00)	272.74 (±9.33)	253.64 (±11.34)

Table S2: Mol % (± standard deviation) of detected amino acids in total hydrolysable dissolved amino acids (THDAA) in the exometabolome of *P. inhibens* DSM 17395 wild type and the two mutants, *tdaE*$^-$ and *pgaR*$^-$ during different growth phases. Concentration of valine in all samples and of phenylalanine in the T_0 samples could not be determined (nd) by HPLC analysis due to interference from a large unknown peak.

**Strain**	**Time point**	**Growth phase**	**Mol %**													
			Asp	Glu	His	Ser	Arg	Gly	Thre	ß-Ala	Ala	Tyr	Val	Phe	Ile	Leu
Wild type (WT)	T_0	Inoculation	80.1 (±13.1)	0	0	0	0	0	9.4 (±18.0)	0	10.5 (±1.0)	0	nd	nd	0	0
	T_1	Lag	9.9 (±0.6)	28.6 (±1.4)	0.4 (±0.02)	0.5 (±0.0)	4.0 (±0.04)	15.8 (±0.5)	14.9 (±0.0)	2.2 (±0.0)	20.4 (±0.2)	2.3 (±0.1)	nd	0.5 (±0.0)	0.5 (±0.0)	0
	T_2	Mid-exponential	6.6 (±0.1)	15.1 (±1.0)	1.2 (±0.4)	4.9 (±0.0)	4.1 (±0.1)	20.2 (±0.3)	7.8 (±1.2)	1.8 (±0.0)	23.14 (±0.4)	4.6 (±0.3)	nd	5.8 (±0.4)	2.4 (±0.0)	2.2 (±0.2)
	T_3	Early stationary	8.8 (±0.8)	13.0 (±0.2)	2.0 (±0.0)	5.6 (±0.0)	4.4 (±0.6)	20.6 (±1.3)	8.2 (±0.6)	0.9 (±0.0)	18.6 (±1.1)	4.1 (±0.5)	nd	4.0 (±0.1)	3.2 (±0.1)	6.5 (±0.5)
tdaE$^-$	T_0	Inoculation	45.1 (±10.5)	52.1 (±10.3)	0	0	1.4 (±2.9)	0	1.4 (±0.9)	0	0	0	nd	nd	0	0
	T_1	Lag	4.3 (±0.3)	39.0 (±0.3)	0	0	2.6 (±0.8)	4.8 (±0.0)	7.2 (±0.2)	4.6 (±8.0)	15.1 (±1.2)	0.3 (±0.0)	nd	10.7 (±0.3)	0	11.5 (±0.1)
	T_2	Mid-exponential	9.2 (±0.2)	11.9 (±1.1)	1.5 (±0.0)	5.3 (±0.4)	3.8 (±0.0)	22.7 (±0.9)	9.4 (±1.0)	0.4 (±0.0)	18.8 (±0.0)	4.0 (±0.4)	nd	4.1 (±0.1)	3.3 (±0.0)	5.6 (±0.1)
	T_3	Early stationary	7.1 (±0.5)	16.9 (±0.1)	1.4 (±0.0)	5.2 (±0.0)	4.6 (±0.1)	17.4 (±0.2)	6.7 (±1.0)	0.1 (±0.2)	24.0 (±0.1)	4.5 (±0.6)	nd	4.1 (±0.2)	2.9 (±0.3)	5.1 (±0.0)
pgaR$^-$	T_0	Inoculation	20.6 (±1.2)	20.2 (±1.1)	0.3 (±0.6)	3.1 (±0.6)	43.9 (±5.4)	7.1 (±0.4)	1.3 (±2.6)	0	1.8 (±0.1)	1.6 (±0.0)	nd	nd	0	0
	T_1	Lag	10.9 (±0.5)	16.0 (±1.2)	1.9 (±0.1)	4.8 (±0.9)	4.3 (±0.0)	17.7 (±0.4)	12.1 (±0.3)	1.6 (±0.1)	18.1 (±0.0)	2.9 (±0.1)	nd	3.0 (±0.4)	3.0 (±0.3)	3.7 (±0.4)
	T_2	Mid-exponential	8.3 (±0.1)	14.2 (±0.6)	1.8 (±0.0)	5.6 (±0.0)	3.9 (±0.1)	18.0 (±0.0)	8.1 (±0.9)	0.2 (±0.0)	25.8 (±0.4)	3.4 (±0.2)	nd	3.4 (±0.3)	2.6 (±0.0)	4.6 (±0.2)
	T_3	Early stationary	8.5 (±1.2)	15.1 (±0.0)	2.0 (±0.2)	5.8 (±0.1)	4.5 (±0.4)	14.6 (±0.2)	6.9 (±0.8)	0.4 (±0.0)	23.8 (±0.0)	4.2 (±0.1)	nd	3.6 (±0.3)	3.4 (±0.0)	7.3 (±0.0)

Table S4: Normalized relative abundance of masses corresponding to tropodithietic acid (TDA) and *N*-3-hydroxydecanoyl-L-homoserine lactone (AHL) at lag (T_1), mid-exponential (T_2) and early stationary (T_3) growth phases of *P. inhibens* DSM 17395 wild type (WT), *tdaE*$^-$ mutant and *pgaR*$^-$ mutant strains. Values are given as mean ± (standard error). TDA was not detected at T_1 for the WT. Relative abundance of TDA showed a $\log_2$ fold change of -1.35 from T_2 to T_3 for the WT. Relative abundance of the AHL showed a $\log_2$ fold change of -2.38, -1.96 and +0.69 for the WT, *tdaE*$^-$ and *pgaR*$^-$, respectively, from T_1 to T_2. Relative abundance of the AHL showed a $\log_2$ fold change of -0.33, -1.17 and -0.64 for the WT, *tdaE*$^-$ and *pgaR*$^-$, respectively, from T_2 to T_3.

m/z	Formula	Metabolite	Bacterial strain	Normalized relative abundance		
				T_1	T_2	T_3
210.953	$C_8H_4O_3S_2$	Tropodithietic acid	Wild type	-	4021 (±313.46)	1573 (±208.03)
270.171	$C_{14}H_{25}NO_4$	*N*-3-hydroxydecanoyl-L-homoserine lactone	Wild type	436 (±162.58)	84 (±10.51)	67 (±11.18)
			tdaE$^-$	682 (±109.94)	175 (±37.24)	78 (±24.16)
			pgaR$^-$	385 (±92)	620 (±115.25)	397 (±37.14)
